# The relationship between self-perceived fatigue, muscle endurance, and circulating markers of inflammation in participants of the Copenhagen aging and Midlife Biobank (CAMB)

**DOI:** 10.1186/s11556-024-00336-9

**Published:** 2024-01-31

**Authors:** Ivan Bautmans, Veerle Knoop, Ingo Beyer, Helle Bruunsgaard, Drude Molbo, Erik Lykke Mortensen, Rikke Lund

**Affiliations:** 1 Gerontology department (GERO), Vrije Universiteit Brussel, Laarbeeklaan 103, Brussel, B-1090 Belgium; 2 Frailty in Ageing Research Group (FRIA), Vrije Universiteit Brussel, Laarbeeklaan 103, Brussel, B-1090 Belgium; 3 Department of Geriatrics, Universitair Ziekenhuis Brussel, Laarbeeklaan 101, Brussel, B-1090 Belgium; 4 SOMT University of Physiotherapy, Softwareweg 5, Amersfoort, 3821 The Netherlands; 5 Section of Social Medicine, Department of Public Health, University of Copenhagen, Copenhagen, Denmark; 6 Section of Environmental Health, Department of Public Health, University of Copenhagen, Copenhagen, Denmark; 7 Center for Healthy Aging, University of Copenhagen, Copenhagen, Denmark; 8 Department of Clinical Immunology, Center for Inflammation and Metabolism, National University Hospital, Copenhagen, Denmark; 9 Institute of Preventive Medicine, Bispebjerg Hospital, Copenhagen, Denmark

**Keywords:** Frailty, Muscle fatigue, Ageing, Handgrip strength, Grip work

## Abstract

**Background:**

Fatigue, low muscle endurance, muscle weakness and low-grade inflammation are strongly related to frailty at higher age. When signs of self-perceived fatigue and low muscle endurance are interrelated with low-grade inflammation at midlife, they might be used as early markers for frailty. This study investigated whether the interrelationships among self-perceived fatigue, muscle endurance and inflammation can be observed at midlife.

**Methods:**

A total of 965 participants of the Copenhagen Aging and Midlife Biobank (aged 52 ± 4 years, 536 males, 426 females) were assessed for self-perceived fatigue (20-item multidimensional fatigue inventory), muscle endurance (grip work), circulating markers of inflammation (hsCRP, IL-6, IL-10, TNF-alpha and IFN-γ), daily physical activity (PAS-2), body composition (%body fat assessed by bio-impedance) and self-reported health status. Participants were categorised (correcting for age and gender) according to high fatigue and/or low muscle endurance, differences in inflammatory profile between fatigue categories were assessed by ANCOVA (corrected for PAS-2, %body fat and presence of inflammatory conditions).

**Results:**

Overall, muscle endurance, fatigue and inflammatory markers were significantly interrelated. Higher levels of hsCRP (*p* < 0.001), IL-6 (*p* < 0.001), IL-10 (*p* = 0.035) and TNF-alpha (*p* = 0.028) were observed in participants presenting both low muscle endurance and high fatigue. IFN-γ was highest in those with high fatigue but normal muscle endurance (*p* = 0.015).

**Conclusions:**

Middle-aged participants with higher fatigue in combination with low muscle endurance show higher levels of inflammation, independently from physical activity, body fat and inflammatory pathology. The underlying mechanisms should be identified and future studies should also investigate whether these individuals show early signs of reduced physiological reserve capacity, which in later life come to full expression by means of frailty.

**Supplementary Information:**

The online version contains supplementary material available at 10.1186/s11556-024-00336-9.

## Background

Fatigue is a common reported complaint among older adults and is increasingly acknowledged as a geriatric syndrome [1]. It poses a significant risk for adverse health outcomes, such as disability, falls, hospitalization and mortality in older adults [2]. Research has shown that muscle strength declines with age and contributes significantly (i.e. more than 20%) to the association between fatigue and physical function in older adults [3]. Sufficiently high maximal strength is crucial for the ability to perform functional tasks including rising from a chair or lifting objects. However, in older persons, continuing and completing these tasks necessitates sustained muscle contractions at a nearly maximal intensity. Reduced muscle endurance might therefore explain the occurrence of fatigue, one of the key characteristics of physical frailty [4, 5]. Fatigue is often considered a marker of aged-related accumulation of deficits, reflecting the underlying vulnerability of an individual’s homeostatic reserves, aligning with the concept of frailty [6]. In the pathophysiology of frailty and fatigue, inflammation has been recognized to play a predominant role in older adults [7], however none of the studies has showed this relationship already at midlife.

Aging is accompanied by a chronic low-grade inflammatory profile (CLIP), characterised by disbalanced levels of pro-inflammatory (hsCRP, IL-6, IL-1β, TNF) and anti-inflammatory (IL-10, IL-1RA) circulating molecules [8–11]. CLIP contributes to sarcopenia [12, 13] (age-related muscle wasting) [14] and frailty [15–20], increasing the risk of negative health outcomes by up to 11-fold [21, 22]. Age-related changes in the immune system, defined as immunosenescence, are also associated with negative health outcomes. Here, late-stage differentiated CD8 + T-cells can become “*inflammescent*” [23], characterized by a senescence-associated secretory phenotype (SASP) [24], leading to exacerbation of CLIP [17, 24–27]. CLIP has also been shown to induce neuroinflammation, increasing the activation of glial cell in the temporal cortex and activity in multiple brain regions in young, middle aged and older adults [28–30] which can lead to centrally stimulated fatigue [31].

Fatigue is one of the main characteristics of frailty, half of the existing frailty scales include at least one fatigue component. It appears that self-perceived fatigue is prominently represented in the frailty scales, while muscle endurance is underrepresented [32]. However, muscle endurance is a parameter that can distinguish frail older adults from their non-frail counterparts [33], therefore muscle endurance is a very promising parameter to help elucidating the pathways leading to frailty as well as to develop preventive and therapeutic interventions. Inflammation seems to have a mediating role in the presence of fatigue in frailty. In addition, a prospective study with participants aged 24–91 years showed that participants with high inflammatory response after abdominal surgery showed higher feelings of self-perceived fatigue and low muscle endurance compared to those with low inflammatory response after surgery [34].

It can be hypothesized that when individuals show already at midlife development of CLIP which would be accompanied by reduced physiological reserve capacity (characterized by low muscle endurance and high feelings of self-perceived fatigue), they might be more prone to physical frailty in the future. This hypothesis is supported by previous research showing that pre-frail older adults had higher levels of self-perceived fatigue combined with low muscle endurance compared to their robust counter parts, which was influenced by inflammatory markers [35]. In older adults, fatigue, muscle endurance and inflammation have been playing a predominant role in the development of frailty [36]. Muscle endurance and self-perceived fatigue are interrelated, however, these factors also provide complementary information about fatigue in adults [34, 37, 38]. In clinical decision making, it can be relevant to know whether older adults experiencing both self-perceived fatigue and low muscle endurance are also those who present CLIP already at midlife in order to target them with preventive interventions to avoid progression towards frailty at later life. Therefore, the aim of this study was to explore the relationship between self-perceived fatigue, muscle endurance, and low-grade inflammation in adults at midlife. In addition, we aimed to investigate whether subgroups can be identified showing at midlife early signs of reduced muscle endurance and/or increased self-perceived fatigue, and to portray their inflammatory status.

## Materials and methods

### Participants

This study included 965 participants, belonging to a subgroup of the Copenhagen Aging and Midlife Biobank (CAMB), which has been described extensively elsewhere [39, 40]. In summary, CAMB is based on a merger of three established cohorts: The Copenhagen Perinatal Cohort (CPC; 8102 men and women born at the National University Hospital in Copenhagen in 1959-61), The Metropolit Cohort (MC; 10,171 men born in Copenhagen in 1953), and the Danish Longitudinal study on Work, Unemployment and Health (DALWUH; 11,082 men and women, born 1949 and 1959, constituting a random sample of the Danish population in 1999). Sample size calculation using G-power [41] indicated a minimum sample size of 103 participants needed for an F-test with 7 predictors to detect a medium effect size (ES = 0.15) with 80% power and alpha = 0.05. For the purpose of the present study the fatigue resistance test, a measure of muscle endurance was added to the test battery [42] in a subset of 965 participants of the cohort who were consecutively enrolled between November 2010 and March 2011. The participants’ enrolment for this study has been described in detail by De Dobbeleer et al. [43]. The local ethical committee has approved the CAMB cohorts (No: H-A-2008-126). CAMB has also been registered at the Danish Data Protection Agency as a combined database (No: 2008-41-2938). All participants provided written informed consent.

### Muscle endurance

Muscle endurance was measured using a Dynamometer G100 system (Biometrics Ltd, Newport, UK), consisting in an adjustable handgrip handle (standard JAMAR dynamometer configuration) equipped with in-build compression load cell (capacity = 0-90 kg, accuracy = < 1% of rated load) and connected via a strain gauge amplifier (National Instruments, type SCC-SG24) to a computer. All tests were performed with the dominant hand. Prior to the start the system was calibrated by applying a 20 kg load to the dynamometer handle and before each assessment the system was adjusted for environmental conditions (ambient temperature) by doing a zero-calibration. To avoid in-between assessment zero-calibrations, the handgrip handle was calibrated in position 2 (middle grip position) and maintained in that position for all participants. Grip strength, fatigue resistance and grip work of the dominant hand were assessed as described previously [38]. Briefly, the subject was asked to squeeze the handle as hard as possible for 3 times with 30 s interval. If the force of the third attempt exceeded both preceding measures by more than 5%, participants performed a fourth attempt. A fifth test was done if the force of the fourth test exceeded the third with more than 5%. The highest of 3–5 attempts was noted as the maximal grip strength (in kg). Afterwards, the subject was again instructed to squeeze the handgrip handle as hard as possible and to maintain this maximal pressure as long as possible. The time (in seconds) during which grip strength dropped to 50% of its maximum was recorded as fatigue resistance [42]. Grip work, a parameter reflecting the total effort produced during the fatigue resistance test, was estimated as described previously [37, 38, 43]. This parameter represents the physiologic work delivered by the handgrip muscles during the fatigue resistance test. Grip Work is calculated by multiplying fatigue resistance in seconds by 75% of the maximal grip strength: (Grip work = 0.75 * maximal grip strength * fatigue resistance). Fatigue resistance and grip work were also expressed per kilogram body mass as described previously [34, 37, 38, 44, 45].

### Self-perceived fatigue

Self-perceived fatigue was assessed using the Danish version of the Multidimensional Fatigue Inventory (MFI-20) [46], covering five domains of fatigue: General Fatigue, Physical Fatigue, Mental Fatigue, Reduced Motivation and Reduced Activities. Each subscale includes four items with five-point categories, resulting in a subscale score range of 4 to 20, with higher scores indicating greater fatigue. According to Watt et al. [46], each subscale was constructed by summation of its four items, if at least half of the items were not missing. Missing items were substituted by the mean of the non-missing items. Finally, also a total MFI-score (score range from 20 to 100) was calculated by summing the scores of the 5 subscales.

### Physical activity

Physical activity was evaluated by the PAS-2 questionnaire and expressed in 24-hour MET score according to Andersen et al. [47]. Briefly, weighed daily hours and minutes of sleep (0.9 MET/hour), TV/sedentary leisure activity (1 MET), Sedentary work (1.5 MET), standing/walking work (2 MET), heavy physical work (5 MET) and commuter cycling/walking (4 MET) were multiplied by 5 for work and commuter activities or by 7 for sleep and TV/sedentary leisure time to obtain MET-time per week. In addition, weighed weekly hours and minutes of light (3 MET), moderately strenuous (5 MET) and strenuous (6 MET) leisure time physical activities were obtained. When the sum of the hours/week reported in the questionnaire was less than 168 h (i.e. 24 h * 7 days), the unreported hours were weighed with a MET-value of 2 MET. Weekly MET-time for all activities were summed and the total weekly MET-time was divided by 7 to calculate the daily summed MET-time. Weekly hours that were recorded in excess (i.e. >168 h) in the questionnaire were divided by 7, weighed with a MET-value of 2 MET and subtracted from the daily MET-time.

### Anthropometry & body composition

Weight and height were measured (at the nearest 0.1 kg and 0.01 m respectively) and body mass index (BMI) was calculated as weight*height^−2^ (kg*m^−2^). Whole body fat was estimated by a Tanita MC-180 bioimpedance (hand-to-foot) device (Tanita corp., Tokyo, Japan) and expressed as percentage of body weight.

### Prevalence of inflammatory conditions

Based on self-reported health status and the interview at the study clinic the presence of (1) acute systemic inflammatory event(s) (i.e. fever, cold, flu, pneumonia, digestive tract infection, urinary tract infection or any other infection during the last 3 weeks; fractures or surgery during the last month; visit at the dentist during the last week), (2) auto-immune disease or arthritis (i.e. chronic intestinal inflammation, arthritis or other connective tissue disease), (3) lung disease (i.e. asthma, chronic bronchitis, emphysema or other serious lung disease) and (4) chronic viral infection (i.e. HIV positive or hepatitis B or C) was recorded.

### Inflammatory mediators

Non-fasting blood samples were taken by venepunction the day of physical testing, before participating in the physical tests. Blood samples were centrifuged at room temperature immediately after sampling and plasma was stored at -80 °C for ulterior (within maximum 2 years) determination of inflammatory biomarkers. High-sensitivity C-reactive protein (hs-CRP) was quantified by immune-turbidimetric analysis by using Roche/Hitachi automatic instrument COBAS®. The assay used was Tina quant (Roche Diagnostics GmbH, Mannheim, Germany) according to the manufacturer’s instructions. Intra-assay and inter-assay precision expressed as coefficient of variance was determined by the manufacturer as respectively 1.34% and 5.70% for low, and 0.28% and 2.51% for high standards; detection limit 0.03 mg/L. Concentrations of IL-6, TNFα, IFNγ and IL-10 were determined simultaneously using an electro-chemiluminescence’s multiplex system on Sector 2400 Imager from Meso Scale Discovery (Gaithersburg, USA) according to the manufactures instructions. Lower limits of detection (LOD) were respectively 0.210pg/mL, 0.281pg/mL, 0.189pg/mL and 0.210pg/mL for IL-6, TNFα, IFNγ and IL-10. We imputed values from a uniform distribution (between 0 and LOD) when measurements of the cytokines fell below the LOD [48].

### Statistical analysis

Analyses were done using IBM SPSS statistics 22 (IBM Corporation, New York, USA). Average values are presented with standard deviation (SD) or quartile deviation (QD, P75-P25) depending on measure level and normality of distribution. Several data presented non-normal distribution as evaluated using the Kolmogorov-Smirnov Goodness of Fit Test (hs-CRP, weekly physical activity) or were scored on an ordinal scale (20–100 and 4–20 for MFI-20 total score and subscale scores respectively). For not normally distributed data as well as for the ordinal variables non-parametric tests were used for analysis. Gender differences were explored with either independent samples t-test or Mann-Whitney U-test. Differences between 3 or more subgroups were assessed by one-way Analysis of Variance (ANOVA) or Kruskall-Wallis test. Bonferroni post-hoc test was performed for post-hoc testing. Spearman’s rho correlation coefficient was computed to explore potential relationships between muscle endurance and self-perceived fatigue, mobility, and inflammation. Grip Work was corrected for body weight and computed for males and females separately because the relation between muscle endurance and inflammatory markers might be influenced by body composition [34, 44, 49]. To test the hypothesis whether persons with low muscle endurance and high feelings of self-perceived fatigue are more prone to inflammation, we assessed the interaction between muscle endurance and self-perceived fatigue (Grip work * 1/MFI-20) on inflammation (Appendix A Table A.2-A.7). Inflammation predicted significantly on all these fatigue interactions. A low score on the MFI-20 and a high score on the grip work are considered to be good, a classic interaction computation as the product of these parameters would neutralize the linear increase in interaction score with combined worsening scores on muscle endurance and self-perceived fatigue. Therefore, we recomputed the MFI-20 scores as 1/MFI-20 for testing its interaction with grip work. In line with earlier research [35] a “Capacity to Perceived Vitality” (CPV) ratio between Grip Work and MFI-20 score was computed (expressed as: Grip Work/ MFI-20), resulting in high ‘combined’ fatigue levels when the ratio was low, and low ‘combined’ fatigue levels when the ratio is high. In total 5 different ratios were computed, CPV_total faigue_, CPV_general fatigue_, CPV_physical fatigue_, CPV_reduced activity_, CPV_reduced motivation_ and CPV_mental fatigue_. Linear regression models were computed with the ratio as dependent factor, and inflammatory markers as independent parameters. Cohort, age, sex, weekly physical activity [24-hour MET score], percentage body fat and presence of inflammatory conditions were used as covariates. To investigate whether the relationships found in the linear regression analyses were biased by the presence of inflammatory conditions we also performed the same analyses for persons with and without inflammatory conditions separately. Additionally, participants were classified according to high or normal levels of self-perceived fatigue (MFI-20 scores) and low or normal levels of muscle endurance (Grip Work), considering values ≥ P70 as high and values ≤ P30 as low. Cutoff-values related to these percentiles were calculated (see supplementary table A.20) and classification of the participants was performed for each cohort (MC, DALWUH, CPC) and sex (males & females) separately. Differences in inflammatory profile were explored according to the combination of self-perceived fatigue and muscle endurance by Analysis of Co-Variance (ANCOVA, with weekly physical activity [24-hour MET score], percentage body fat and presence of inflammatory conditions as covariates) and accounted for multiple-testing with the Bonferroni post-hoc test correction. Non-normal distributed inflammatory parameters were log10-transformed and studentized residuals obtained by the corrected models were verified for normality of distribution using the Kolmogorov-Smirnov Goodness of Fit Test. Differences in sex and cohort with categorisation according to the combination of self-perceived fatigue and muscle endurance were analysed by Chi-Square test. Significance was set a priori at two-sided *p* < 0.05.

## Results

The participants’ characteristics are shown in Table 1 and in Appendix A table A.1. There was no significant difference between the three cohorts for body weight, BMI and body fat. Physical activity level as well as maximal grip strength were significantly higher in CPC participants compared to MC and (female) DALWUH participants. Muscle endurance was similar in the three cohorts, except for uncorrected grip work, which was significantly higher in male CPC compared to MC participants. Males showed significantly higher body weight, grip strength and grip work, as well as lower body fat and BMI (only among CPC) compared to females. Overall, self-perceived fatigue was significantly higher in MC participants compared to DALWUH (total, physical and mental fatigue, and reduced activity) and CPC (all subscales) participants. Among the CPC participants, females showed significantly higher fatigue scores (total, general and physical fatigue) compared to males. There was a significant difference in CPV ratio’s between the three cohorts; the males in the MC cohort scored significantly lower compared to the CPC male participants on all ratios. The overall prevalence of inflammatory pathology (acute and chronic) was 57.4% (expressed as the percentage of the number of participants with an inflammatory condition relative to the total number of participants) without significant differences between cohorts or sex, except for the presence of auto-immune disease or osteoarthritis which was significantly lower in the male DALWUH compared to MC and CPC male participants. Regarding the inflammatory parameters, MC showed significantly higher IL-6 levels compared to (male) CPC participants, and TNF-alpha was significantly higher in male versus female CPC participants.

**Table 1:** Participants’ characteristics

Parameter	MC	DALWUH		CPC	
	Males	Males	Females	Males	Females
*Physical characteristics*	*n* = 175	*n* = 41	*n* = 59	*n* = 320	*n* = 370
Age (years)	57.9 ± 0.3^a, b^	56.6 ± 5.1^b^	56.8 ± 5.1^b^	50.1 ± 0.8	50.1 ± 0.8
Height (m)	1.792 ± 0.067	1.777 ± 0.085	1.653 ± 0.063^b^	1.800 ± 0.064	1.672 ± 0.060
Weight (kg)	85.7 ± 14.7	83.7 ± 14.3^c^	71.5 ± 14.4	86.9 ± 15.0^c^	71.1 ± 14.1
Body Mass Index (kg*m^−2^)	26.7 ± 4.1	26.5 ± 4.2	26.1 ± 4.8	26.8 ± 4.3^c^	25.4 ± 4.9
Body Fat (%)	22.6 ± 5.8	21.4 ± 6.7^c^	32.5 ± 6.8	21.3 ± 6.3^c^	31.4 ± 6.9
Physical activity (24-hour MET score)	38.8 (36.9–42.4)^b^	39.8 (37.3–44.6)	38.6 (36.5–40.7)^b^	40.1 (37.4–43.3)	39.3 (37.7–42.2)
Maximal Grip Strength (kg)	48.4 ± 8.0^b^	50.4 ± 7.6^c^	31.1 ± 5.4^b^	52.3 ± 8.6^c^	32.6 ± 5.1
Fatigue Resistance (sec)	33.4 (24.3–44.6)	35.2 (26.9–46.1)	33.7 (20.4–48.6)	34.7 (27.0-47.2)	36.4 (25.5–53.7)
Grip Work (kg*sec)	1264.8^b^ (874.7-1625.6)	1287.7 (983.2-1858.3)	814.2 (467.0-1085.3)	1375.9^c^ (1028.2-1841.7)	897.1 (600.4-1266.3)
Grip Work /body mass (kg*sec*kg^−1^)	14.4 (10.5–19.5)	16.4 (11.9–21.5)^c^	11.5 (5.7–18.2)	16.4 (11.6–21.8)^c^	12.4 (8.0-19.3)
*Self-perceived fatigue (MFI-20)*	*n* = 159	*n* = 37	*n* = 56	*n* = 311	*n* = 366
Total Fatigue score (score 20–100)	46 (36–55)^a, b^	38 (31–45)	39 (31–53)	36 (30–46)^c^	41 (31–52)
CPV ratio	*n* = 159	*n* = 37	*n* = 56	*n* = 311	*n* = 366
CPV- Total Fatigue ratio	28,58^b^ (19,93 − 40,98)	32,36^c^ (22,82 − 54,67)	17,97 (12,61 − 31,73	38,39^c^ (26,05–51,62)	22,07 (12,49 − 35,67)
*Inflammatory conditions(y/n)*	*115/60*	*22/19*	*36/23*	*179/140*	*201/168*
Acute systemic inflammatory event (y/n)	74/101	15/26	24/35	118/201	134/235
Auto-immune disease or arthritis (y/n)	55/120	8/33	16/43	66/254	87/283
Lung disease (y/n)	27/148	5/36	7/52	37/283	47/323
Chronic viral infection (y/n)	0/175	1/40	0/59	3/316	2/367
*Inflammatory parameters*	*n* = 169	*n* = 41	*n* = 59	*n* = 316	*n* = 363
hs-CRP (mg/L)	1.0 (0.5–2.3)	1.0 (0.5–2.5)	1.2 (0.5–2.4)	1.1 (0.5–2.3)	0.9 (0.5–2.1)
IFN-γ (pg/mL)	0.43 (0.30–0.63)	0.44 (0.32–0.81)	0.45 (0.34–0.58)	0.40 (0.28–0.66)	0.40 (0.28–0.58)
IL-6 (pg/mL)	2.01 (1.37–3.55)^b^	1.85 (1.16–3.01)	1.86 (1.27–2.65)	1.47 (1.01–2.28)	1.46 (1.02–2.34)
IL-10 (pg/mL)	1.29 (0.80–2.06)	1.34 (0.84–2.98)	1.00 (0.60–2.22)	1.05 (0.65–1.88)	0.91 (0.53–1.79)
TNF-alpha (pg/mL)	4.74 (4.02–6.02)	5.19 (4.46–6.24)	4.22 (3.56–5.40)	4.58 (3.38–5.59)^c^	4.13 (3.47–5.22)

Values expressed as mean ± SD for continuous variables, and as median (P25-P75) for ordinal variables and continuous variables with not-normal distribution; MC = Metropolitan Cohort; DALWUH = Danish Longitudinal Study on Work Unemployment and Health; CPC = Copenhagen Perinatal Cohort; y = yes, *n* = no; significantly different from ^a^DALWUH, ^b^CPC (One-way ANOVA with Bonferroni post hoc test for males & unpaired t-test for females for continuous variables, Mann-Whitney U test for ordinal variables, *p* < 0.05); ^c^significantly different from females participants within same cohort (unpaired t-test for continuous variables, Mann-Whitney U test for ordinal variables, *p* < 0.05), ^d^significantly different from MC and CPC (Chi-Square test *p* < 0.05)

As can be seen in Table 2, higher muscle endurance was significantly related to less self-perceived fatigue (except for general fatigue and fatigue resistance). When stratified for sex, grip work was significantly related to all self-perceived fatigue subscales except for general and mental fatigue in the male, whereas for muscle endurance the relationship with the subscales reduced activity (males), reduced motivation (males) and mental fatigue (males & females) lost statistical significance. Higher age was significantly associated to higher self-perceived fatigue (except for mental fatigue) and lower physical activity levels. In the male participants separately, all relationships remained statistically significant, whereas in the females age remained only significantly related to the level of physical activity and the fatigue subscale reduced activity. Higher level of physical activity was significantly related to better muscle endurance and grip work.

**Table 2:** Relationships between muscle endurance, self-perceived fatigue, age and physical activity

Parameter	Age			Fatigue Resistance^a^			Grip Work^a^		
	Males	Females	Total	Males	Females	Total	Males	Females	Total
Age	-	-	-	0.02	-0.08	-0.07*	-0.05	-0.10*	-0.02
Total Fatigue	**0.19**^**†**^	0.06	**0.11**^**†**^	-0.09*	**-0.17**^**†**^	**-0.12**^**†**^	-0.10*	**-0.21**^**†**^	**-0.16**^**†**^
General Fatigue	**0.16**^**†**^	0.03	0.08*	-0.04	-0.09	-0.05	-0.05	-0.12*	**-0.10**^**†**^
Physical Fatigue	**0.21**^**†**^	0.04	**0.10**^**†**^	-0.10*	**-0.23**^**†**^	**-0.13**^**†**^	-0.11*	**-0.26**^**†**^	**-0.20**^**†**^
Reduced Activity	**0.16**^**†**^	0.10*	**0.13**^**†**^	-0.08	**-0.21**^**†**^	**-0.15**^**†**^	-0.09*	**-0.25**^**†**^	**-0.16**^**†**^
Reduced motivation	**0.12**^**†**^	0.04	**0.09**^**†**^	-0.08	**-0.13**^**†**^	**-0.10**^**†**^	-0.09*	**-0.17**^**†**^	**-0.12**^**†**^
Mental Fatigue	0.08	0.05	0.06	-0.06	-0.08	-0.07*	-0.05	-0.11*	-0.08*
Physical activity	-0.10*	**-0.14**^**†**^	**-0.11**^**†**^	0.09*	0.11*	**0.10**^**†**^	0.11*	0.12*	**0.13**^**†**^

^a^Corrected for body weight, values represent Spearman Rho correlation coefficients *p < 0.05, ^†^ *p* < 0.01

In Table 3 the relationships of inflammatory markers with age, self-perceived fatigue, physical activity and muscle endurance are shown. Higher age was significantly related to higher circulating cytokine levels (IFN-γ, IL-6, IL-10 and TNF-alpha), but not with hs-CRP. Overall, higher levels of self-perceived fatigue were significantly related to higher circulating levels of hs-CRP and IL-6, except for mental fatigue (for hs-CRP in the males and for IL-6 in both sexes separately). IFN-γ was only significantly related to total fatigue (only in females) and with the subscales physical fatigue (only in females) and reduced activity (not in males and females separately). Higher levels of IL-10 were significantly related to higher scores on the subscales reduced motivation and mental fatigue (both scales not in male separately), and with reduced activity (not in male and female separately). Higher TNF-alpha levels were significantly related to all fatigue subscales (except for mental fatigue only in females and for general fatigue), but not in the male separately (except for total fatigue and reduced activity). Low muscle endurance was significantly related to higher levels of circulating hs-CRP, IL-6 and TNF-alpha (except for fatigue resistance in males), whereas no significant relationships were found with circulating IFN-γ or IL-10.

**Table 3:** Relationships of inflammation with age, self-perceived fatigue, physical activity and muscle endurance

Parameter	hs-CRP			IFN-γ			IL-6			IL-10			TNF-alpha		
	Males	Females	Total	Males	Females	Total	Males	Females	Total	Males	Females	Total	Males	Females	Total
Age	0.03	0.07	0.06	0.10*	**0.14**^**†**^	**0.13**^†^	**0.26**^**†**^	0.12*	**0.21**^**†**^	**0.16**^**†**^	0.12*	**0.16**^**†**^	**0.17**^**†**^	**0.17**^**†**^	**0.20**^**†**^
Total Fatigue	**0.19**^**†**^	**0.24**^**†**^	**0.21**^**†**^	0.03	0.10*	0.06	**0.29**^**†**^	**0.26**^**†**^	**0.27**^**†**^	0.06	0.06	0.05	0.09*	**0.17**^**†**^	**0.12**^**†**^
General Fatigue	0.11*	0.12*	**0.11**^**†**^	0.03	0.06	0.04	**0.23**^**†**^	**0.14**^**†**^	**0.18**^**†**^	0.08	-0.01	0.04	0.07	0.09	0.06
Physical Fatigue	**0.24**^**†**^	**0.33**^**†**^	**0.27**^**†**^	0.03	0.11*	-0.02	**0.31**^**†**^	**0.32**^**†**^	**0.30**^**†**^	0.01	-0.02	-0.02	0.08	**0.14**^**†**^	0.08*
Reduced Activity	**0.19**^**†**^	**0.22**^**†**^	**0.20**^**†**^	0.06	0.08	0.07*	**0.29**^**†**^	**0.30**^**†**^	**0.29**^**†**^	0.07	0.07	0.07*	**0.13**^**†**^	**0.21**^**†**^	**0.17**^**†**^
Reduced motivation	**0.12**^**†**^	**0.15**^**†**^	**0.14**^**†**^	0.02	0.08	0.05	**0.14**^**†**^	**0.17**^**†**^	**0.16**^**†**^	0.04	0.10*	0.07*	0.05	**0.13**^**†**^	**0.10**^**†**^
Mental Fatigue	0.03	0.13*	0.08*	-0.03	0.06	0.02	0.08	0.10	**0.09**^**†**^	0.01	0.13*	0.07*	-0.02	**0.14**^**†**^	0.05
Physical activity	0.01	-0.11*	-0.04	0.02	0.02	0.02	-0.09	-0.12*	**-0.10**^**†**^	-0.05	0.03	-0.01	-0.05	-0.06	-0.04
Fatigue Resistance^a^	**-0.15**^**†**^	**-0.24**^**†**^	**-0.19**^**†**^	0.01	-0.08	-0.04	**-0.11**^**†**^	**-0.22**^**†**^	**-0.17**^**†**^	0.01	-0.05	-0.04	-0.06	-0.10*	**-0.11**^**†**^
Grip Work^a^	**-0.18**^**†**^	**-0.26**^**†**^	**-0.21**^**†**^	-0.01	-0.09	-0.04	**-0.17**^†^	**-0.25**^**†**^	**-0.19**^**†**^	-0.04	-0.06	-0.03	-0.10*	-0.11*	-0.07*

^a^Corrected for body weight, values represent Spearman Rho correlation coefficients *p < 0.05, ^†^ *p* < 0.01

**Fig. 1:**
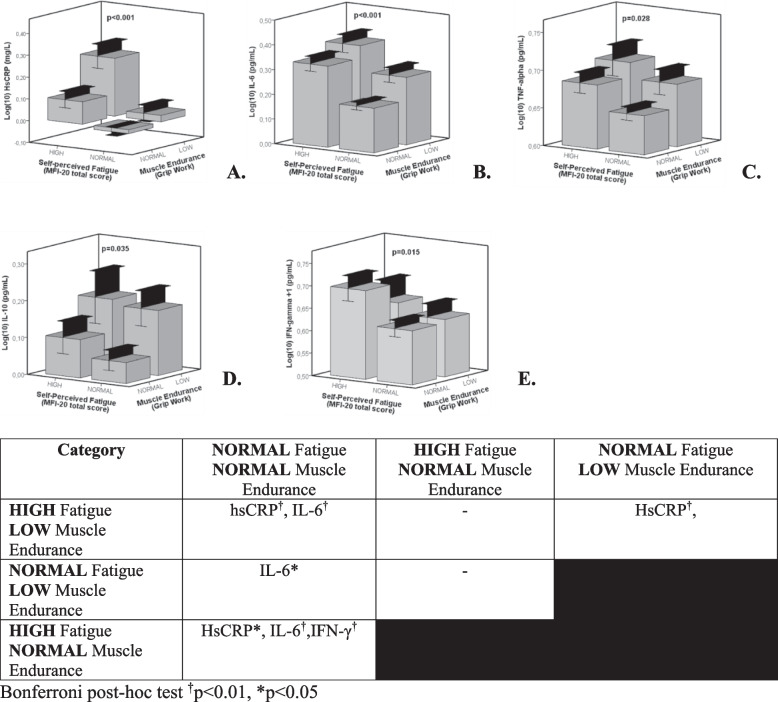
Circulating levels of inflammatory mediators according to self-perceived fatigue and muscle endurance. Bars represent mean ± standard error. Inflammatory markers are expressed as log(10) transformed values. Since all log(10) transformed values For IFN-γ were negative (IFN-γ concentrations were >1) all data were summed by 1 in order to optimise the visual interpretation of figure 1E. ANCOVA analysis (with weekly physical activity, percentage body fat and presence of inflammatory conditions as covariates) revealed significant differences between the subgroups for **A** hs-CRP (*p*<0.001), **B** IL-6 (*p*<0.001), **C** TNF-alpha (*p*=0.028), **D** IL-10 (*p*=0.035) and **E** IFN-γ (*p*=0.015). Overall, participants with high self-perceived fatigue and low muscle endurance presented the highest (except for IFN-γ, figure 1E) and those with normal self-perceived fatigue and normal muscle endurance the lowest levels of inflammatory mediators. **F** Post-hoc pairwise comparisons showed that participants with high self-perceived fatigue and low muscle endurance presented significantly higher hs-CRP and IL-6 levels compared to those with normal self-perceived fatigue and normal muscle endurance (*p*<0.01), as well as lower hs-CRP compared to those with normal self-perceived fatigue but low muscle endurance (*p*<0.01). Participants with normal self-perceived fatigue and normal muscle endurance showed also significantly lower levels of IL-6 compared to those with normal self-perceived fatigue and low muscle endurance (p<0.05), and lower hs-CRP, IL-6 and IFN-γ compared to participants with high self-perceived fatigue and normal muscle endurance (respectively*p*<0.05, *p*<0.01 and *p*<0.01)

Significant relationships of inflammatory markers with the CPV ratios (ratio Grip work/MFI, reflecting the interplay between muscle endurance and self-perceived fatigue) were found (Appendix A table A.2-A.7). For IL-6 and hs-CRP, there was a significant relationship with all CPV ratio’s. Also the other inflammatory markers were significantly related to several CPV ratio’s (except for TNF-alpha with CPV_physical fatigue_, CPV_general fatigue_ and CPV_mental fatigue_; for IFN-γ with CPV_reduced activity_ and CPV_reduced motivation_ ; and for IL-10 with CPV_physical fatigue_ and CPV_general fatigue_). Since the presence of inflammatory conditions was a significant predictor for the CPV-ratios, we have repeated the linear regression analyses for participants with and without inflammatory conditions separately (see supplementary tables A.8 t/m A.19). Overall, the inflammatory biomarkers showed higher (standardized) beta coefficients in subjects without compared to those with inflammatory conditions. For IL-6 and CRP, the association with the CPV ratios remained statistically significant in participants with and without inflammatory conditions (except for CPV-mental which was significantly predicted by these biomarkers in subjects without inflammatory conditions contrary to those with inflammatory conditions, and for CPV general where the association with HsCRP was borderline (*p* = 0.052) significant in those without inflammatory conditions). For IL-10, IFN-γ and TNF-alpha the association with the CPV ratios lost statistical significance in subjects with inflammatory conditions (except for the relation between IL-10 and CPV_general fatigue_) whereas it remained statistically significant in those without inflammatory conditions for IL-10 with CPV_reduced activity_, IFN-ɣ with all CPV ratios and TNF-alpha with CPV_general fatigue_. In a next step, participants were classified according to high or normal levels of self-perceived fatigue and low or normal levels of muscle endurance (considering values ≥ P70 as high and values ≤ P30 as low). No significant differences in sex or cohort between categories according to the combination of muscle endurance and self-perceived fatigue were found (Chi-Square test, all p-values > 0.05 for each fatigue subscale). As can be seen in Fig. 1, participants with high self-perceived fatigue and low muscle endurance presented significantly the highest levels of inflammatory markers (except for IFN-γ) where those with high self-perceived fatigue and normal muscle endurance showed the highest levels, see Fig. 1E) and those with normal self-perceived fatigue and normal muscle endurance the lowest levels of inflammatory markers (all ANCOVA analyses *p* < 0.05). As shown in Table 4, a similar pattern was found when considering the MFI-20 subscales for self-perceived fatigue (all ANCOVA analyses *p* < 0.05 except for TNF-alpha on the subscales ‘general fatigue’ and ‘reduced motivation’ and for IFN-γ on all subscales).

**Table 4:** Circulating levels of inflammatory mediators according to self-perceived fatigue and muscle endurance

Parameter	HIGH Fatigue LOW Muscle Endurance	NORMAL Fatigue NORMAL Muscle Endurance	HIGH Fatigue NORMAL Muscle Endurance	NORMAL Fatigue LOW Muscle Endurance	*p*-value*
General Fatigue	*n* = 119	*n* = 431	*n* = 225	*n* = 154	
Log hsCRP (pg/mL)	0.213 ± 0.42^a^	0.015 ± 0.022^b^	0.040 ± 0.030^b^	0.008 ± 0.036^b^	< 0.001
Log IL-6 (pg/mL)	0.348 ± 0.032^a^	0.193 ± 0.017^b^	0.280 ± 0.023^a^	0.260 ± 0.032	< 0.001
Log TNF-α (pg/mL)	0.695 ± 0.018	0.654 ± 0.009	0.667 ± 0.013	0.685 ± 0.016	0.137
Log IL-10 (pg/mL)	0.156 ± 0.057	0.064 ± 0.030	0.068 ± 0.042	0.214 ± 0.05	0.043
Log IFN-γ (pg/mL)	-0.346 ± 0.036	-0.389 ± 0.019	-0.320 ± 0.026	-0.396 ± 0.036	0.130
Physical Fatigue	*n* = 102	*n* = 454	*n* = 202	*n* = 171	
Log hsCRP (pg/mL)	0.184 ± 0.046^a^	-0.016 ± 0.021^b^	0.109 ± 0.032^a^	0.049 ± 0.034	< 0.001
Log IL-6 (pg/mL)	0.353 ± 0.035^a^	0.191 ± 0.016^b^	0.293 ± 0.025^a^	0.268 ± 0.026	< 0.001
Log TNF-α (pg/mL)	0.688 ± 0.020	0.649 ± 0.009	0.679 ± 0.014	0.690 ± 0.020	0.046
Log IL-10 (pg/mL)	0.160 ± 0.063	0.091 ± 0.030	0.009 ± 0.044	0.204 ± 0.047^c^	0.018
Log IFN-γ (pg/mL)	-0.364 ± 0.040	-0.387 ± 0.019	-0.317 ± 0.028	-0.379 ± 0.030	0.220
Reduced Activity	*n* = 113	*n* = 438	*n* = 218	*n* = 160	
Log hsCRP (pg/mL)	0.182 ± 0.043^a^	-0.015 ± 0.021^b^	0.100 ± 0.031^a^	0.039 ± 0.035	< 0.001
Log IL-6 (pg/mL)	0.363 ± 0.033^a^	0.184 ± 0.016^b^	0.300 ± 0.024^a^	0.254 ± 0.027	< 0.001
Log TNF-α (pg/mL)	0.709 ± 0.018^a^	0.641 ± 0.009^b^	0.694 ± 0.013^a^	0.677 ± 0.015	0.001
Log IL-10 (pg/mL)	0.207 ± 0.059	0.050 ± 0.030	0.097 ± 0.043	0.178 ± 0.049	0.037
Log IFN-γ (pg/mL)	-0.347 ± 0.037	-0.381 ± 0.019	-0.334 ± 0.027	-0.393 ± 0.031	0.388
Reduced Motivation	*n* = 121	*n* = 436	*n* = 220	*n* = 152	
Log hsCRP (pg/mL)	0.159 ± 0.041^a^	-0.008 ± 0.022^b^	0.085 ± 0.031	0.047 ± 0.037	0.002
Log IL-6 (pg/mL)	0.316 ± 0.031^a^	0.186 ± 0.017^b^	0.296 ± 0.023^a^	0.284 ± 0.028^a^	< 0.001
Log TNF-α (pg/mL)	0.696 ± 0.018	0.650 ± 0.009	0.677 ± 0.013	0.684 ± 0.016	0.052
Log IL-10 (pg/mL)	0.194 ± 0.057	0.038 ± 0.030	0.119 ± 0.042	0.187 ± 0.051	0.019
Log IFN-γ (pg/mL)	-0.341 ± 0.036	-0.384 ± 0.019	-0.329 ± 0.027	-0.401 ± 0.032	0.228
Mental Fatigue	*n* = 104	*n* = 467	*n* = 189	*n* = 168	
Log hsCRP (pg/mL)	0.190 ± 0.044^a^	-0.001 ± 0.021^b^	0.083 ± 0.033	0.038 ± 0.035^b^	0.001
Log IL-6 (pg/mL)	0.263 ± 0.034	0.189 ± 0.016	0.307 ± 0.025^a^	0.318 ± 0.027^a^	< 0.001
Log TNF-α (pg/mL)	0.685 ± 0.019	0.649 ± 0.009	0.684 ± 0.014	0.691 ± 0.015	0.031
Log IL-10 (pg/mL)	0.141 ± 0.061	0.032 ± 0.029	0.150 ± 0.046	0.216 ± 0.048^a^	0.005
Log IFN-γ (pg/mL)	-0.383 ± 0.039	-0.382 ± 0.018	-0.324 ± 0.029	-0.369 ± 0.030	0.373

ANCOVA analysis (with weekly physical activity, percentage body fat and presence of inflammatory conditions as covariates); values are expressed as adjusted mean ± SE; ^a^significantly different from participants with NORMAL Fatigue & NORMAL Muscle Endurance (Bonferroni post hoc test *p* < 0.05); ^b^significantly different from participants with HIGH Fatigue & LOW Muscle Endurance. (Bonferroni post hoc test *p* < 0.05); ^c^significantly different from participants with HIGH Fatigue & NORMAL Muscle Endurance (Bonferroni post hoc test *p* < 0.05)

## Discussion

This study aimed at exploring the interrelationship among muscle endurance, self-perceived fatigue and circulating levels of inflammatory markers at midlife. The main finding was that middle-aged participants with high self-perceived fatigue in combination with low muscle endurance presented the highest levels of inflammatory markers. These results are in line with earlier research, in a prospective study involving abdominal surgery patients aged 24–91 years, we found that worsening muscle endurance after surgery was significantly related to higher IL-6 release following surgery and to higher self-perceived fatigue following the intervention. Surgery-induced increase in circulating IL-6 at day 4 post-surgery was highest in patients showing both worsened muscle endurance and worsened self-perceived fatigue [34]. Our observations were significant for hs-CRP, IL-6, IL-10 and TNF-α and were independent from age, sex, co-morbidity, body composition and physical activity level since classification of the participants according to high or normal levels of self-perceived fatigue and low or normal levels of muscle endurance was performed for each cohort and sex separately. All analysis were corrected for the presence of inflammatory conditions. In addition, we found that the associations between inflammatory biomarkers and CPV ratios were stronger (i.e. more consistent statistical significance and showing higher standardized beta coefficients) in persons without compared to those with inflammatory conditions. This supports our hypothesis that the association between increased inflammatory biomarkers and low CPV ratios might be due to CLIP and not disease-induced inflammation.

Surprisingly, we observed that the anti-inflammatory cytokine IL-10 followed the same pattern as the pro-inflammatory biomarkers when looking at the association between fatigue and inflammation. However, the association between IL-10 and fatigue is less pronounced compared to the pro-inflammatory biomarkers. Based on previous research it is plausible that also the level of IL-10 slightly increases with CLIP, since during ongoing pro-inflammatory activity anti-inflammatory mechanisms increase slightly [50–52]. IL-10 is an anti-inflammatory cytokine that typically acts to downregulate the inflammatory response and to prevent excessive inflammation. However, in certain situations, elevated IL-10 levels may also be associated with immune system activation. It is possible that the immune system is attempting to counterbalance the pro-inflammatory effects by increasing anti-inflammatory signals. This compensatory mechanism might therefore induce a small increase in IL-10 during CLIP [51].

Intriguingly, for IFN-γ, the highest levels were seen in those participants with high self-perceived fatigue (total MFI-score) in combination with normal muscle endurance. Likewise, when considering the MFI subscales, no significant differences were observed between the four groups for IFN-γ levels. In the literature, increased IFN-γ levels, inducing increased tryptophan degradation and enhanced neopterin formation, have been associated with self-perceived fatigue symptoms in patients with symptomatic Epstein-Barr virus infection [53], and with depressive mood and fatigue in older persons with CLIP [54]. Possibly, IFN-γ mediated fatigue is a different type of fatigue with another underlying mechanism than that evaluated in our study participants. Another interesting observation is the fact that the separate relationships between muscle endurance and self-perceived fatigue on the one hand and the relationships with inflammatory mediators on the other hand were - although statistically significant - rather moderate to low (Spearman Rho correlation coefficients between +/-0.07 and +/-0.23). In future studies addressing the role of midlife self-perceived fatigue and muscle endurance on frailty at older age, the evaluation of both parameters should be continued, as they are not interchangeable.

The impact of inflammation on self-perceived fatigue has been demonstrated earlier. In fact, inflammatory cytokines released in the peripheral blood circulation can on the one hand cross the blood-brain barrier, causing sickness behaviour and on the other hand activate specific immune-to-brain communication pathways (such as vagal nerve stimulation), thus affecting dopaminergic, serotonine and norepinephrine neurotransmission in the central nervous system, and induce fatigue sensations [55, 56]. Literature on the relationship between self-perceived fatigue and inflammation in apparently healthy, community-dwelling adults are more limited compared to evidence from studies involving patients suffering from inflammatory diseases [57] (such as cancer, neurological conditions and auto-immune disease). In our study we showed that when performing the analysis for persons with and without inflammatory conditions separately, the relationship between CPV ratios and inflammatory markers was even higher in the persons without inflammatory conditions, providing evidence that our findings were not biased by disease-related inflammation. Other studies showed higher levels of fatigue in relation to higher circulating levels of inflammatory biomarkers in middle-aged diabetes patients (aged 52 ± 14 years) [58] and middle-aged breast cancer survivors (aged 55 ± 8 years) [59]. On the other hand, two prospective studies found no significant relationship between changes in self-perceived fatigue [60] and trajectories of vital exhaustion [61] during young adulthood and circulating markers of inflammation at early midlife. However, participants of the Coronary Artery Risk Development in Young Adults study with persistently higher levels of CRP at early mid-life showed a significantly higher prospective risk for the occurrence of self-perceived fatigue over 5-years follow-up [62]. Cooper, Popham [63] found that underweight or obese adults (age 60–64) with higher levels of IL-6 showed higher levels of physical fatigability, in our analysis we corrected for bodyweight and fat and the results remained similar. Similarly, Whitehall II study participants (aged 39–63 years) presenting higher CRP and IL-6 levels showed significantly higher odds for new-onset fatigue at 3 years follow-up (OR = 1.28 [1.09–1.49] for high CRP and OR = 1.24 [1.06–1.45] for high IL-6) [64]. Even though we have performed a cross sectional study, these data suggest a causal relationship between low-grade inflammation at middle-age and the subsequent development or worsening of self-perceived fatigue.

Self-perceived fatigue is mostly related to central factors while muscle endurance is mediated by central as well as peripheral factors (or a combination of both) [65]. Central factors increasing muscle endurance include reduced efferent supraspinal drive of motoneurons and direct inhibition of motoneurons due to altered afferent input from muscle receptors [66, 67]. Peripheral factors – at the level of the muscle itself - contributing to muscle fatigue include alterations of the actin-myosin interactions, excitation-contraction coupling and sarcoplasmic reticulum function [67]. The pathways through which inflammation affects skeletal muscle fatigue have been described in an extensive review by Morris et al. [68] However, muscle fatigue is rarely assessed in clinical studies. Previously, we showed that inflammation impairs recovery of muscle endurance in hospitalised geriatric patients, despite medical treatment of the aetiology and physiotherapy [69]. In another study we found evidence for the involvement of peripheral processes in the loss of muscle endurance in hospitalized geriatric patients with acute inflammation [70]. In the present study we cannot exclude that both central and peripheral factors were involved explaining the inflammation-related self-perceived fatigue and low muscle endurance. However, there is evidence that both types of fatigue can be influenced by central processes. Research showed that increased levels of inflammatory cytokines affect neuroinflammation [28–30] which can lead to fatigue. Ho, Teresi [71] reported that levels of IL-6 are associated with neuroinflammation in young adolescents with depression while Vints, Kušleikiene [28] found a significant relationship between elevated levels of serum kynurenine and neuroinflammation in older adults. Therefore, it can not be excluded that the interplay between peripheral and central fatigue was mediated by inflammation.

To our knowledge, this is one of the first reports on the relationship between muscle endurance and low-grade inflammation in a population of community-dwelling middle-aged adults. Moreover, literature data regarding the impact of inflammation on combined self-perceived and muscle fatigue are extremely scarce. Based on the results of our present study we suggest that older adults showing higher sensations of fatigue in combination with lower muscle endurance might represent a clinical subgroup with reduced physiological reserve capacity. It can be hypothesized that this subgroup of individuals is at higher risk for the development of physical frailty at higher age. However, our results and hypothesis need to be confirmed and validated in future prospective studies. The underlying factors for the significantly higher levels of inflammatory biomarkers in the CAMB-participants presenting high self-perceived fatigue in combination with low muscle endurance remain unclear. In fact, low-grade inflammation has been associated to various factors including age, body fat, physical inactivity as well as to comorbidity [26, 72]. However, in our study we have adjusted the data for age and sex, and corrected statistical analyses for age, sex, physical activity, percentage body fat and inflammatory pathology. It can be hypothesized that these older adults show already early signs of CLIP or immunosenescence. Although CLIP and immunosenescence are usually considered as characteristics of older adults at high age (i.e. >65 years), it is likely that this results from insidious and progressive processes starting already earlier in life. Latent viral infection such as CMV is considered to accelerate the accumulation of senescent immune cells which are assumed to be involved in the occurrence of CLIP [73, 74]. Unfortunately, we have no data regarding CMV seropositivity in our participants. Depression is another factor that can contribute the interrelationship between fatigue and inflammation, but that was not included in our analysis. Higher levels of pro-inflammatory markers such as CRP, IL-6 and TNF-α and more fatigue are seen in patients with depressive disorders [75, 76] because of negative effects on the nervous system [77].

The strength of this study relies in the inclusion of a relatively large sample of CAMB participants, a representative cohort of middle-aged participants living in and around Copenhagen (DK), in whom psychological (self-perceived fatigue), physical (muscle endurance) and biological (inflammatory biomarkers) outcomes were assessed; providing unique data on potentially early determinants of frailty. Another strength is the fact that all data and statistical analyses were adjusted and corrected for potential confounding factors. However, this study has also some limitations. Firstly, information on health status and medication use was based on self-report and interview, and it cannot be excluded that some clinical conditions might have been under-reported or missed. Secondly, we have no data on latent viral infection or proportions of T-cells showing senescence markers of our participants; and thus the underlying causes of low-grade inflammation in those participants with high fatigue and low muscle endurance remains speculative. Finally, this is a cross-sectional study design and our results need to be confirmed in future prospective research.

## Conclusion

We found that middle-aged older adults with higher fatigue in combination with lower muscle endurance show higher levels of inflammation, independently from age, sex, physical activity, body fat and inflammatory pathology. The underlying mechanisms should be identified, and future studies should also investigate whether these individuals show early signs of reduced physiological reserve capacity, which at later life might come to full expression as frailty.

### Supplementary Information

**Additional file 1: Table A.1.** Participants’ characteristics Subscores MFI-20 and CPV ratio. **Table A.2.** Linear regression on CPV- total ratio. **Table A.3.** Linear regression on CPV- general fatigue ratio. **Table A.4.** Linear regression on CPV- physical fatigue ratio. **Table A.5.** Linear regression CPV- reduced activity ratio.**Table A.6.** Linear regression on CPV- reduced motivation ratio. **Table A.7.** Linear regression on CPV- mental fatigue ratio. **Table A.8.** Linear regression on CPV- total ratio for per persons with inflammatory conditions. **Table A.9.** Linear regression on CPV- total ratio for per persons without inflammatory conditions. **Table A.10.** Linear regression on CPV- general ratio for per persons with inflammatory conditions. **Table A.11.** Linear regression on CPV- general ratio for per persons without inflammatory conditions. **Table A.12.** Linear regression on CPV- physical ratio for per persons with inflammatory conditions. **Table A.13.** Linear regression on CPV- physical ratio for per persons without inflammatory conditions. **Table A.14.** Linear regression on CPV- reduced activation ratio for per persons with inflammatory conditions. **Table A.15.** Linear regression on CPV- reduced activation ratio for per persons without inflammatory conditions. **Table A.16.** Linear regression on CPV- reduced motivation ratio for per persons with inflammatory conditions. **Table A.17.** Linear regression on CPV- reduced motivation ratio for per persons without inflammatory conditions. **Table A.18.** Linear regression on CPV- mental fatigue ratio for per persons with inflammatory conditions. **Table A.19.** Linear regression on CPV- mental fatigue ratio for per persons without inflammatory conditions. **Table A.20.** Gender -and cohort-specific cutoff values used for HIGH self-perceived fatigue and LOW muscle endurance.

## Data Availability

All data generated or analyzed during this study are included in this article or its supplementary material files. Further enquiries can be directed to the corresponding authors.

## References

[1] Liao S, Ferrell BA (2000). Fatigue in an older population. J Am Geriatr Soc.

[2] Knoop V, Cloots B, Costenoble A, Debain A, Azzopardi RV, Vermeiren S (2021). Fatigue and the prediction of negative health outcomes: a systematic review with meta-analysis. Ageing Res Rev.

[3] Manty M, de Leon CF, Rantanen T, Era P, Pedersen AN, Ekmann A (2012). Mobility-related fatigue, walking speed, and muscle strength in older people. J Gerontol A Biol Sci Med Sci.

[4] Fried LP, Tangen CM, Walston J, Newman AB, Hirsch C, Gottdiener J (2001). Frailty in older adults: evidence for a phenotype. J Gerontol A Biol Sci Med Sci.

[5] Avlund K, Damsgaard MT, Sakari-Rantala R, Laukkanen P, Schroll M (2002). Tiredness in daily activities among nondisabled old people as determinant of onset of disability. J Clin Epidemiol.

[6] Fried LP, Tangen CM, Walston J, Newman AB, Hirsch C, Gottdiener J (2001). Frailty in older adults: evidence for a phenotype. J Gerontol A Biol Sci Med Sci.

[7] Bauer JM, Sieber CC (2008). Sarcopenia and frailty: a clinician’s controversial point of view. Exp Gerontol.

[8] Krabbe KS, Pedersen M, Bruunsgaard H (2004). Inflammatory mediators in the elderly. Exp Gerontol.

[9] Minciullo PL, Catalano A, Mandraffino G, Casciaro M, Crucitti A, Maltese G (2016). Inflammaging and anti-inflammaging: the role of cytokines in Extreme Longevity. Arch Immunol Ther Exp (Warsz).

[10] Morrisette-Thomas V, Cohen AA, Fülöp T, Riesco É, Legault V, Li Q (2014). Inflamm-aging does not simply reflect increases in pro-inflammatory markers. Mech Ageing Dev.

[11] Calder PC, Bosco N, Bourdet-Sicard R, Capuron L, Delzenne N, Dore J (2017). Health relevance of the modification of low grade inflammation in ageing (inflammageing) and the role of nutrition. Ageing Res Rev.

[12] Tuttle CSL, Thang LAN, Maier AB (2020). Markers of inflammation and their association with muscle strength and mass: a systematic review and meta-analysis. Ageing Res Rev.

[13] Wahlin-Larsson B, Carnac G, Kadi F (2014). The influence of systemic inflammation on skeletal muscle in physically active elderly women. Age.

[14] Cruz-Jentoft AJ, Bahat G, Bauer J, Boirie Y, Bruyere O, Cederholm T (2019). Sarcopenia: revised European consensus on definition and diagnosis. Age Ageing.

[15] Welstead M, Muniz-Terrera G, Russ TC, Corley J, Taylor AM, Gale CR (2020). Inflammation as a risk factor for the development of frailty in the Lothian Birth Cohort 1936. Exp Gerontol.

[16] Álvarez-Satta M, Berna-Erro A, Carrasco-Garcia E, Alberro A, Saenz-Antoñanzas A, Vergara I (2020). Relevance of oxidative stress and inflammation in frailty based on human studies and mouse models. Aging.

[17] Ferrucci L, Fabbri E (2018). Inflammageing: chronic inflammation in ageing, cardiovascular disease, and frailty. Nat Rev Cardiol.

[18] Sayed N, Huang Y, Nguyen K, Krejciova-Rajaniemi Z, Grawe AP, Gao T (2021). An inflammatory aging clock (iAge) based on deep learning tracks multimorbidity, immunosenescence, frailty and cardiovascular aging. Nat Aging.

[19] Landino K, Tanaka T, Fantoni G, Candia J, Bandinelli S, Ferrucci L. Characterization of the plasma proteomic profile of frailty phenotype. GeroScience. 2021;43(2):1029–37. 10.1007/s11357-020-00288-9 PMC8110642 33200349

[20] Fried LP, Cohen AA, Xue Q-L, Walston J, Bandeen-Roche K, Varadhan R (2021). The physical frailty syndrome as a transition from homeostatic symphony to cacophony. Nat Aging.

[21] Vermeiren S, Vella-Azzopardi R, Beckwee D, Habbig AK, Scafoglieri A, Jansen B (2016). Frailty and the prediction of negative Health outcomes: a Meta-analysis. J Am Med Dir Assoc.

[22] Su Y-C, Chang S-F, Tsai H-C (2022). The relationship between Sarcopenia and Injury events: a systematic review and Meta-analysis of 98,754 older adults. J Clin Med.

[23] Morris SR, Chen B, Mudd JC, Panigrahi S, Shive CL, Sieg SF (2020). Inflammescent CX3CR1 + CD57 + CD8 + T cells are generated and expanded by IL-15. JCI Insight.

[24] Akbar AN, Henson SM, Lanna A (2016). Senescence of T lymphocytes: implications for Enhancing Human immunity. Trends Immunol.

[25] Pawelec G, Bronikowski A, Cunnane SC, Ferrucci L, Franceschi C, Fülöp T (2020). The conundrum of human immune system “senescence”. Mech Ageing Dev.

[26] Pawelec G, Goldeck D, Derhovanessian E (2014). Inflammation, ageing and chronic disease. Curr Opin Immunol.

[27] Tchkonia T, Zhu Y, Van Deursen J, Campisi J, Kirkland JL (2013). Cellular senescence and the senescent secretory phenotype: therapeutic opportunities. J Clin Invest.

[28] Vints WAJ, Kušleikiene S, Sheoran S, Šarkinaite M, Valatkevičiene K, Gleizniene R (2022). Inflammatory blood biomarker kynurenine is linked with elevated neuroinflammation and neurodegeneration in older adults: evidence from two 1H-MRS post-processing analysis methods. Front Psychiatry.

[29] Lind A, Boraxbekk CJ, Petersen ET, Paulson OB, Andersen O, Siebner HR (2021). Do glia provide the link between low-grade systemic inflammation and normal cognitive ageing? A (1) H magnetic resonance spectroscopy study at 7 tesla. J Neurochem.

[30] Lind A, Boraxbekk CJ, Petersen ET, Paulson OB, Siebner HR, Marsman A (2020). Regional Myo-Inositol, Creatine, and choline levels are higher at older Age and Scale negatively with Visuospatial Working Memory: a cross-sectional Proton MR Spectroscopy Study at 7 Tesla on normal cognitive ageing. J Neurosci.

[31] Karshikoff B, Sundelin T, Lasselin J (2017). Role of inflammation in human fatigue: relevance of Multidimensional assessments and potential neuronal mechanisms. Front Immunol.

[32] Knoop V, Costenoble A, Vella Azzopardi R, Vermeiren S, Debain A, Jansen B (2019). The operationalization of fatigue in frailty scales: a systematic review. Ageing Res Rev.

[33] De Dobbeleer L, Theou O, Beyer I, Jones GR, Jakobi JM, Bautmans I (2018). Martin Vigorimeter assesses muscle fatigability in older adults better than the Jamar Dynamometer. Exp Gerontol.

[34] Bautmans I, Njemini R, De Backer J, De Waele E, Mets T (2010). Surgery-induced inflammation in relation to age, muscle endurance, and self-perceived fatigue. J Gerontol A Biol Sci Med Sci.

[35] Knoop V, Costenoble A, Debain A, Azzopardi RV, Vermeiren S, Laere SV (2021). The interrelationship between grip work, self-perceived fatigue and pre-frailty in community-dwelling octogenarians. Exp Gerontol.

[36] Theou O, Jones GR, Overend TJ, Kloseck M, Vandervoort AA (2008). An exploration of the association between frailty and muscle fatigue. Applied physiology, nutrition, and metabolism = Physiologie appliquee, nutrition et metabolisme.

[37] Bautmans I, Njemini R, Predom H, Lemper JC, Mets T (2008). Muscle endurance in elderly nursing home residents is related to fatigue perception, mobility, and circulating tumor necrosis factor-alpha, interleukin-6, and heat shock protein 70. J Am Geriatr Soc.

[38] Bautmans I, Gorus E, Njemini R, Mets T (2007). Handgrip performance in relation to self-perceived fatigue, physical functioning and circulating IL-6 in elderly persons without inflammation. BMC Geriatr.

[39] Avlund K, Osler M, Mortensen EL, Christensen U, Bruunsgaard H, Holm-Pedersen P (2014). Copenhagen Aging and Midlife Biobank (CAMB): an introduction. J Aging Health.

[40] Lund R, Mortensen EL, Christensen U, Bruunsgaard H, Holm-Pedersen P, Fiehn NE, et al. Cohort profile: the Copenhagen aging and midlife Biobank (CAMB). Int J Epidemiol. 2016;45(4):1044–53. 10.1093/ije/dyv149 26210613

[41] Faul F, Erdfelder E, Lang AG, Buchner A (2007). G*Power: A flexible statistical power analysis program for the social, behavioral, and biomedical sciences. Behavior Research Methods.

[42] Bautmans I, Mets T (2005). A fatigue resistance test for elderly persons based on grip strength: reliability and comparison with healthy young subjects. Aging Clin Exp Res.

[43] De Dobbeleer L, Beyer I, Hansen AM, Molbo D, Mortensen EL, Lund R (2019). Grip work measurement with the Jamar Dynamometer: validation of a simple equation for clinical use. J Nutr Health Aging.

[44] Bautmans I, Onyema O, Van Puyvelde K, Pleck S, Mets T (2011). Grip work estimation during sustained maximal contraction: validity and relationship with dependency and inflammation in elderly persons. J Nutr Health Aging.

[45] Beyer I, Bautmans I, Njemini R, Demanet C, Bergmann P, Mets T (2011). Effects on muscle performance of NSAID treatment with piroxicam versus placebo in geriatric patients with acute infection-induced inflammation. A double blind randomized controlled trial. BMC Musculoskelet Disord.

[46] Watt T, Groenvold M, Bjorner JB, Noerholm V, Rasmussen NA, Bech P (2000). Fatigue in the Danish general population. Influence of sociodemographic factors and disease. J Epidemiol Commun Health.

[47] Andersen LG, Groenvold M, Jorgensen T, Aadahl M (2010). Construct validity of a revised physical activity scale and testing by cognitive interviewing. Scand J Public Health.

[48] Herbers J, Miller R, Walther A, Schindler L, Schmidt K, Gao W (2021). How to deal with non-detectable and outlying values in biomarker research: best practices and recommendations for univariate imputation approaches. Compr Psychoneuroendocrinology.

[49] Ronti T, Lupattelli G, Mannarino E (2006). The endocrine function of adipose tissue: an update. Clin Endocrinol.

[50] Rink L, Cakman I, Kirchner H (1998). Altered cytokine production in the elderly. Mech Ageing Dev.

[51] Álvarez-Rodríguez L, López-Hoyos M, Muñoz-Cacho P, Martínez-Taboada VM (2012). Aging is associated with circulating cytokine dysregulation. Cell Immunol.

[52] Rea IM, Gibson DS, McGilligan V, McNerlan SE, Alexander HD, Ross OA (2018). Age and Age-Related diseases: role of inflammation triggers and cytokines. Front Immunol.

[53] Bellmann-Weiler R, Schroecksnadel K, Holzer C, Larcher C, Fuchs D, Weiss G (2008). IFN-gamma mediated pathways in patients with fatigue and chronic active Epstein Barr virus-infection. J Affect Disord.

[54] Capuron L, Schroecksnadel S, Feart C, Aubert A, Higueret D, Barberger-Gateau P (2011). Chronic low-grade inflammation in elderly persons is associated with altered tryptophan and tyrosine metabolism: role in neuropsychiatric symptoms. Biol Psychiatry.

[55] Dantzer R, Heijnen CJ, Kavelaars A, Laye S, Capuron L (2014). The neuroimmune basis of fatigue. Trends Neurosci.

[56] Dantzer R, Kelley KW (2007). Twenty years of research on cytokine-induced sickness behavior. Brain Behav Immun.

[57] Whitehead LC, Unahi K, Burrell B, Crowe MT (2016). The experience of fatigue across long-term conditions: a qualitative Meta-synthesis. J Pain Symptom Manag.

[58] Lasselin J, Laye S, Dexpert S, Aubert A, Gonzalez C, Gin H (2012). Fatigue symptoms relate to systemic inflammation in patients with type 2 diabetes. Brain Behav Immun.

[59] Orre IJ, Reinertsen KV, Aukrust P, Dahl AA, Fossa SD, Ueland T (2011). Higher levels of fatigue are associated with higher CRP levels in disease-free breast cancer survivors. J Psychosom Res.

[60] Cho HJ, Bower JE, Kiefe CI, Seeman TE, Irwin MR (2012). Early life stress and inflammatory mechanisms of fatigue in the coronary artery Risk Development in Young adults (CARDIA) study. Brain Behav Immun.

[61] Hoekstra T, Barbosa-Leiker C, Twisk JW (2013). Vital exhaustion and markers of low-grade inflammation in healthy adults: the Amsterdam Growth and Health Longitudinal Study. Stress and Health: Journal of the International Society for the Investigation of Stress.

[62] Cho HJ, Seeman TE, Bower JE, Kiefe CI, Irwin MR (2009). Prospective association between C-reactive protein and fatigue in the coronary artery risk development in young adults study. Biol Psychiatry.

[63] Cooper R, Popham M, Santanasto AJ, Hardy R, Glynn NW, Kuh D (2019). Are BMI and inflammatory markers independently associated with physical fatigability in old age?. Int J Obes (Lond).

[64] Cho HJ, Kivimaki M, Bower JE, Irwin MR (2013). Association of C-reactive protein and interleukin-6 with new-onset fatigue in the Whitehall II prospective cohort study. Psychol Med.

[65] Enoka RM, Duchateau J (2008). Muscle fatigue: what, why and how it influences muscle function. J Physiol.

[66] Gandevia SC (2001). Spinal and supraspinal factors in human muscle fatigue. Physiol Rev.

[67] Kent-Braun JA, Fitts RH, Christie A (2012). Skeletal muscle fatigue. Compr Physiol.

[68] Morris G, Berk M, Galecki P, Walder K, Maes M (2016). The Neuro-Immune pathophysiology of central and peripheral fatigue in systemic Immune-Inflammatory and Neuro-Immune diseases. Mol Neurobiol.

[69] Bautmans I, Njemini R, Lambert M, Demanet C, Mets T (2005). Circulating Acute Phase mediators and skeletal muscle performance in hospitalized geriatric patients. J Gerontol A Biol Sci Med Sci.

[70] Arnold P, Njemini R, Vantieghem S, Duchateau J, Mets T, Beyer I (2017). Peripheral muscle fatigue in hospitalised geriatric patients is associated with circulating markers of inflammation. Exp Gerontol.

[71] Ho TC, Teresi GI, Segarra JR, Ojha A, Walker JC, Gu M (2021). Higher levels of pro-inflammatory cytokines are Associated with higher levels of glutamate in the Anterior Cingulate Cortex in Depressed adolescents. Front Psychiatry.

[72] Franceschi C, Capri M, Monti D, Giunta S, Olivieri F, Sevini F (2007). Inflammaging and anti-inflammaging: a systemic perspective on aging and longevity emerged from studies in humans. Mech Ageing Dev.

[73] Soderberg-Naucler C, Fornara O, Rahbar A (2016). Cytomegalovirus driven immunosenescence-An immune phenotype with or without clinical impact?. Mech Ageing Dev.

[74] Solana R, Tarazona R, Aiello AE, Akbar AN, Appay V, Beswick M (2012). CMV and immunosenescence: from basics to clinics. Immun Ageing.

[75] Miller AH, Haroon E, Raison CL, Felger JC (2013). Cytokine targets in the brain: impact on neurotransmitters and neurocircuits. Depress Anxiety.

[76] Penninx BWJH, Kritchevsky SB, Yaffe K, Newman AB, Simonsick EM, Rubin S (2003). Inflammatory markers and depressed mood in older persons: results from the health, aging and body composition study. Biol Psychiatry.

[77] Felger JC, Miller AH (2012). Cytokine effects on the basal ganglia and dopamine function: the subcortical source of inflammatory malaise. Front Neuroendocrinol.

