# Supplementary material for: The relationship between self-perceived fatigue, muscle endurance, and circulating markers of inflammation in participants of the Copenhagen aging and Midlife Biobank (CAMB)
Source: Eur Rev Aging Phys Act. 2024 Jan 31;21:2. doi: 10.1186/s11556-024-00336-9 (PMC10829210; doi:10.1186/s11556-024-00336-9)
Supplement: Supplementary file 1 — Additional file 1: Table A.1. Participants’ characteristics Subscores MFI-20 and CPV ratio. Table A.2. Linear regression on CPV- total ratio. Table A.3. Linear regression on CPV- general fatigue ratio. Table A.4. Linear regression on CPV- physical fatigue ratio. Table A.5. Linear regression CPV- reduced activity ratio.Table A.6. Linear regression on CPV- reduced motivation ratio. Table A.7. Linear regression on CPV- mental fatigue ratio. Table A.8. Linear regression on CPV- total ratio for per persons with inflammatory conditions. Table A.9. Linear regression on CPV- total ratio for per persons without inflammatory conditions. Table A.10. Linear regression on CPV- general ratio for per persons with inflammatory conditions. Table A.11. Linear regression on CPV- general ratio for per persons without inflammatory conditions. Table A.12. Linear regression on CPV- physical ratio for per persons with inflammatory conditions. Table A.13. Linear regression on CPV- physical ratio for per persons without inflammatory conditions. Table A.14. Linear regression on CPV- reduced activation ratio for per persons with inflammatory conditions. Table A.15. Linear regression on CPV- reduced activation ratio for per persons without inflammatory conditions. Table A.16. Linear regression on CPV- reduced motivation ratio for per persons with inflammatory conditions. Table A.17. Linear regression on CPV- reduced motivation ratio for per persons without inflammatory conditions. Table A.18. Linear regression on CPV- mental fatigue ratio for per persons with inflammatory conditions. Table A.19. Linear regression on CPV- mental fatigue ratio for per persons without inflammatory conditions. Table A.20. Gender -and cohort-specific cutoff values used for HIGH self-perceived fatigue and LOW muscle endurance. [file 11556_2024_336_MOESM1_ESM.docx]

**APPENDIX A**

**The relationship between self-perceived fatigue, muscle endurance, and circulating markers of inflammation in participants of the Copenhagen Aging and Midlife Biobank (CAMB)**

| Parameter | MC | DALWUH | | CPC | |
| --- | --- | --- | --- | --- | --- |
|  | **Males** | **Males** | **Females** | **Males** | **Females** |
| *Self-perceived fatigue (MFI-20)* | *n=159* | *n=37* | *n=56* | *n=311* | *n=366* |
| Total Fatigue score (score 20-100) | 46 (36-55)^a, b^ | 38 (31-45) | 39 (31-53) | 36 (30-46)^c^ | 41 (31-52) |
| General Fatigue score (score 4-20) | 11 (7-13)^b^ | 10 (7-13) | 9 (7-12) | 8 (6-11)^c^ | 10 (7-13) |
| Physical Fatigue score (score 4-20) | 10 (7-13)^a, b^ | 8 (6-12) | 9 (6-12) | 8 (5-10)^c^ | 9 (6-12) |
| Reduced Activity score (score4-20) | 9 (6-11)^a, b^ | 7 (5-9) | 8 (5-10) | 7 (5-9) | 7 (5-10) |
| Reduced motivation score (score 4-20) | 8 (6-9)^b^ | 7 (6-8) | 7 (5-9) | 7 (5-8) | 7 (5-10) |
| Mental Fatigue score (score 4-20) | 8 (6-11)^a, b^ | 6 (4-8) | 7 (5-9) | 7 (5-9) | 7 (5-10) |
| CPV ratio | *n=159* | *n=37* | *n=56* | *n=311* | *n=366* |
| CPV- Total Fatigue ratio | 28,58^b^  (19,93-40,98) | 32,36^c^  (22,82-54,67) | 17,97  (12,61-31,73 | 38,39^c^  (26,05-51,62) | 22,07  (12,49-35,67) |
| CPV- General Fatigue ratio | 123,38^b^  (83,39-195,06) | 143,18^c^  (89,65-219,61) | 77,10  (55,95-116,85) | 167,67^c^  (119,09-257,81) | 91,60  (53,68-153,99) |
| CPV- Physical Fatigue ratio | 135,29^b^  (83,15-192,85) | 158,21^c^  (114,48-218,62) | 88,90  (47,13-169,75) | 188,71^c^  (117,27-278,54) | 96,60  (54,59-166,60) |
| CPV- Reduced Activity ratio | 150,80^b^  (1003,35-222,07) | 185,40^c^  (121,15-293,35) | 92,37  (59,55-175,64) | 200,87^c^  (131,98-299,28) | 123,72  (68,03-209,06) |
| CPV-Reduced motivation ratio (score 4-20) | 175,93^b^  (112,29-236,76) | 185,08^c^  (136,15-300,39) | 102,48  (71,96-196,64) | 210,09^c^  (140,77-307,40) | 130,27  (78,84-217,52) |
| CPV- Mental Fatigue ratio | 160,38^b^  (106,75-229,31) | 207,87^c^  (148,52-309,99) | 106,12  (76,42-167,31) | 207,80^c^  (139,08-304,32) | 126,82  (68,29-203,77) |

**Table A.1 Participants’ characteristics Subscores MFI-20 and CPV ratio**

Values expressed as mean ± SD for continuous variables, and as median (P25-P75) for ordinal variables and continuous variables with not-normal distribution; MC= Metropolitan Cohort; DALWUH= Danish Longitudinal Study on Work Unemployment and Health; CPC= Copenhagen Perinatal Cohort; y=yes, n=no; significantly different from ^a^DALWUH, ^b^CPC (One-way ANOVA with Bonferroni post hoc test for males & unpaired t-test for females for continuous variables, Mann-Whitney U test for ordinal variables, p<0.05); ^c^significantly different from females participants within same cohort (unpaired t-test for continuous variables, Mann-Whitney U test for ordinal variables, p<0.05), ^d^significantly different from MC and CPC (Chi-Square test p<0.05)

**Table A.2. Linear regression on CPV- total ratio**

| **Variables** | **Unstandardized B** | **S.E.** | **Standardized B** | **Sig.** | **95% confidence interval** |
| --- | --- | --- | --- | --- | --- |
| **Log (10) hs-CRP** | -5.931 | 1.546 | -0.132 | <0.001 | -8.965- -2.897 |
| Cohort | 4.690 | 1.680 | 0.169 | 0.005 | 1.393-7.986 |
| Gender | -12.273 | 1.792 | -0.291 | <0.001 | -15.790- -8.755 |
| Age (years) | 0.152 | 0.329 | 0.027 | 0.644 | -0.494-0.798 |
| Percentage body fat (%) | -0.242 | 0.111 | -0.093 | 0.030 | -0.460- -0.024 |
| Weekly physical activity (24-h MET) | 0.697 | 0.132 | 0.164 | <0.001 | 0.437-0.956 |
| Presence of inflammatory conditions | 3.143 | 1.315 | 0.074 | 0.017 | 0.563-5.724 |
|  |  |  |  |  |  |
| **Log (10) IL-6** | -8.947 | 1.971 | -0.148 | <0.001 | -12.815- -2.079 |
| Cohort | 4.813 | 1.669 | 0.174 | 0.004 | 1.537-8.090 |
| Gender | -11.995 | 1.741 | -0.285 | <0.001 | -15.413- -8.578 |
| Age (years) | 0.290 | 0.328 | 0.052 | 0.378 | -0.355-0.934 |
| Percentage body fat (%) | -0.276 | 0.104 | -0.107 | 0.008 | -0.479- -0.072 |
| Weekly physical activity (24-h MET) | 0.664 | 0.132 | 0.157 | <0.001 | 0.406-0.922 |
| Presence of inflammatory conditions | 3.201 | 1.305 | 0.075 | 0.014 | 0.639-5.764 |
|  |  |  |  |  |  |
| **Log (10) IL-10** | -2.350 | 1.077 | -0.068 | 0.029 | -4.464- -0.236 |
| Cohort | 4.749 | 1.690 | 0.171 | 0.005 | 1.433-8.065 |
| Gender | -10.480 | 1.725 | -0.248 | <0.001 | -13.865- -7.095 |
| Age (years) | 0.183 | 0.331 | 0.033 | 0.580 | -0.466-0.832 |
| Percentage body fat (%) | -0.413 | 0.101 | -0.160 | <0.001 | -0.611- -0.215 |
| Weekly physical activity (24-h MET) | 0.686 | 0.133 | 0.162 | <0.001 | 0.424-0.947 |
| Presence of inflammatory conditions | 3.475 | 1.320 | 0.082 | 0.009 | 0.884-6.065 |
|  |  |  |  |  |  |
| **Log (10) IFN-γ** | -3.356 | 1.702 | -0.061 | 0.049 | -6.696- -0.017 |
| Cohort | 4.954 | 1.688 | 0.179 | 0.003 | 4.641-8.266 |
| Gender | -10.671 | 1.724 | -0.253 | <0.001 | -14.055- -7.286 |
| Age (years) | 0.183 | 0.330 | 0.033 | 0.579 | -0.465-0.832 |
| Percentage body fat (%) | -0.388 | 0.101 | -0.150 | <0.001 | -0.585- -0.190 |
| Weekly physical activity (24-h MET) | 0.697 | 0.133 | 0.165 | <0.001 | 0.436-0.958 |
| Presence of inflammatory conditions | 3.976 | 1.315 | 0.094 | 0.003 | 1.396-6.557 |
|  |  |  |  |  |  |
| **Log (10) TNF-alpha** | -0.7012 | 3.526 | -0.063 | 0.047 | -13.932- -0.092 |
| Cohort | 4.972 | 1.688 | 0.180 | 0.003 | 1.659-8.286 |
| Gender | -11.015 | 1.744 | -0.261 | <0.001 | -14.438- -7.593 |
| Age (years) | 0.218 | 0.332 | 0.039 | 0.512 | -0.433-0.869 |
| Percentage body fat (%) | -0.384 | 0.101 | -0.149 | <0.001 | -0.583- -0.186 |
| Weekly physical activity (24-h MET) | 0.676 | 0.133 | 0.160 | <0.001 | 0.416-0.937 |
| Presence of inflammatory conditions | 3.666 | 1.313 | 0.086 | 0.005 | 1.090-6.243 |

*Linear regression analysis with GW/MFI-20 total score as dependent variable. Sig.: p-value <0.05*

**Table A.3. Linear regression on CPV- general fatigue ratio**

| **Variables** | **Unstandardized B** | **S.E.** | **Standardized B** | **Sig.** | **95% confidence interval** |
| --- | --- | --- | --- | --- | --- |
| **Log (10) Hs-CRP** | -24.206 | 8.390 | -0.101 | 0.004 | -40.672- -7.740 |
| Cohort | 29.508 | 9.117 | 0.200 | 0.001 | 11.613-47.402 |
| Gender | -71.689 | 9.727 | -0.319 | <0.001 | -90.781- -52.597 |
| Age (years) | 1.577 | 1.786 | 0.053 | 0.378 | -1.929-5.082 |
| Percentage body fat (%) | -0.593 | 0.603 | -0.043 | 0.326 | -1.776-0.591 |
| Weekly physical activity (24-h MET) | 2.703 | 0.718 | 0.120 | <0.001 | 1.293-4.113 |
| Presence of inflammatory conditions | 22.378 | 7.135 | 0.099 | 0.002 | 8.373-36.382 |
|  |  |  |  |  |  |
| **Log (10) IL-6** | -44.339 | 10.673 | -0.138 | <0.001 | -35.286- -23.391 |
| Cohort | 30.142 | 9.040 | 0.205 | 0.001 | 12.399-47.885 |
| Gender | -72.565 | 9.430 | -0.323 | <0.001 | -91.073- -54.057 |
| Age (years) | 2.250 | 1.779 | 0.076 | 0.206 | -1.241-5.741 |
| Percentage body fat (%) | -0.598 | 0.561 | -0.044 | 0.286 | -1.699-0.502 |
| Weekly physical activity (24-h MET) | 2.532 | 0.713 | 0.112 | <0.001 | 1.133-3.931 |
| Presence of inflammatory conditions | 22.547 | 7.070 | 0.100 | 0.001 | 8.671-36.423 |
|  |  |  |  |  |  |
| **Log (10) IL-10** | -10.559 | 5.825 | -0.057 | 0.070 | -21.993-0.875 |
| Cohort | 29.875 | 9.139 | 0.202 | 0.001 | 11.937-47.813 |
| Gender | -65.052 | 9.330 | -0.290 | <0.001 | -83.363- -46.741 |
| Age (years) | 1.709 | 1.790 | 0.057 | 0.340 | -1.804-5.222 |
| Percentage body fat (%) | -1.282 | 0.546 | -0.093 | 0.019 | -2.354- -0.210 |
| Weekly physical activity (24-h MET) | 2.639 | 0.720 | .117 | <0.001 | 1.226-4.053 |
| Presence of inflammatory conditions | 23.950 | 7.139 | 0.106 | 0.001 | 9.937-37.962 |
|  |  |  |  |  |  |
| **Log (10) IFN-γ** | -18.476 | 9.194 | -0.063 | 0.045 | -36.521- -0.430 |
| Cohort | 30.847 | 9.119 | 0.209 | 0.001 | 12.948-48.745 |
| Gender | -65.851 | 9.318 | -0.294 | <0.001 | -84.139- -47.563 |
| Age (years) | 1.770 | 1.786 | 0.059 | 0.322 | -1.735-5.275 |
| Percentage body fat (%) | -1.143 | 0.545 | -0.083 | 0.036 | -2.212- -0.074 |
| Weekly physical activity (24-h MET) | 2.724 | 0.718 | 0.121 | <0.001 | 1.315-4.134 |
| Presence of inflammatory conditions | 26.232 | 7.105 | 0.116 | <0.001 | 12.287-40.178 |
|  |  |  |  |  |  |
| **Log (10) TNF-alpha** | -34.275 | 19.058 | -0.058 | 0.072 | -71.679-3.129 |
| Cohort | 30.910 | 9.126 | 0.210 | 0.001 | 12.999-48.821 |
| Gender | -67.442 | 9.425 | -0.301 | <0.001 | -85.941- -48.943 |
| Age (years) | 1.922 | 1.794 | 0.065 | 0.284 | -1.599-5.442 |
| Percentage body fat (%) | -1.133 | 0.546 | -0.083 | 0.038 | -2.205- -0.061 |
| Weekly physical activity (24-h MET) | 2.616 | 0.717 | 0.116 | <0.001 | 1.208-4.023 |
| Presence of inflammatory conditions | 24.634 | 7.095 | 0.109 | 0.001 | 10.708-38.559 |

*Linear regression analysis with GW/MFI-20 general fatigue score as dependent variable. Sig.: p-value <0.05*

**Table A.4. Linear regression on CPV- physical fatigue ratio**

| **Variables** | **Unstandardized B** | **S.E.** | **Standardized B** | **Sig.** | **95% confidence interval** |
| --- | --- | --- | --- | --- | --- |
| **Log (10) hs-CRP** | -34.342 | 8.797 | -0.130 | <0.001 | -51.609- -17.077 |
| Cohort | 34.110 | 9.560 | 0.210 | <0.001 | 15.346-52.874 |
| Gender | -52.571 | 10.200 | -0.212 | <0.001 | -72.590- -32.551 |
| Age (years) | 1.673 | 1.873 | 0.051 | 0.372 | -2.003-5.349 |
| Percentage body fat (%) | -3.250 | 0.632 | -0.214 | <0.001 | -4.491- -2.008 |
| Weekly physical activity (24-h MET) | 4.552 | 0.753 | 0.183 | <0.001 | 3.074-6.031 |
| Presence of inflammatory conditions | 15.009 | 7.482 | 0.060 | 0.045 | 0.324-29.694 |
|  |  |  |  |  |  |
| **Log (10) IL-6** | -52.056 | 11.257 | -0.146 | <0.001 | -74.151- -29.961 |
| Cohort | 34.790 | 9.535 | 0.214 | <0.001 | 16.075-53.505 |
| Gender | -49.996 | 9.946 | -0.202 | <0.001 | -69.518- -30.474 |
| Age (years) | 2.457 | 1.876 | 0.075 | 0.191 | -1.225-6.139 |
| Percentage body fat (%) | -3.493 | 0.591 | -0.231 | <0.001 | 2.893-5.844 |
| Weekly physical activity (24-h MET) | 4.369 | 0.752 | 0.176 | <0.001 | 2.893-5.844 |
| Presence of inflammatory conditions | 14.910 | 7.457 | 0.060 | 0.046 | 0.274-29.546 |
|  |  |  |  |  |  |
| **Log (10) IL-10** | -5.972 | 6.166 | -0.029 | 0.333 | -18.074-6.131 |
| Cohort | 34.571 | 9.674 | 0.212 | <0.001 | 15.584-53.558 |
| Gender | -41.033 | 9.875 | -0.166 | <0.001 | -60.415- -21.651 |
| Age (years) | 1.763 | 1.894 | 0.054 | 0.352 | -1.955-5.481 |
| Percentage body fat (%) | -4.286 | 0.578 | -0.282 | <0.001 | -5.420- -3.151 |
| Weekly physical activity (24-h MET) | 4.474 | 0.762 | 0.180 | <0.001 | 2.978-5.970 |
| Presence of inflammatory conditions | 17.106 | 7.557 | 0.069 | 0.024 | 2.274-31.938 |
|  |  |  |  |  |  |
| **Log (10) IFN-γ** | -20.876 | 9.719 | -0.065 | 0.032 | -39.950- -1.802 |
| Cohort | 35.694 | 9.639 | 0.219 | <0.001 | 16.776-54.613 |
| Gender | -42.382 | 9.849 | -0.171 | <0.001 | -61.713- -23.052 |
| Age (years) | 1.852 | 1.888 | 0.056 | 0.327 | -1.852-5.557 |
| Percentage body fat (%) | -4.138 | 0.576 | -0.273 | <0.001 | -5.268- -3.007 |
| Weekly physical activity (24-h MET) | 4.566 | 0.759 | 0.184 | <0.001 | 3.077-6.055 |
| Presence of inflammatory conditions | 19.513 | 7.510 | 0.078 | 0.010 | 4.773-34.253 |
|  |  |  |  |  |  |
| **Log (10) TNF-alpha** | -29.342 | 20.171 | -0.045 | 0.146 | -68.931-10.247 |
| Cohort | 35.364 | 9.659 | 0.218 | <0.001 | 16.407-54.322 |
| Gender | -43.403 | 9.976 | -0.175 | <0.001 | -62.982- -23.823 |
| Age (years) | 1.927 | 1.899 | 0.059 | 0.310 | -1.799-5.654 |
| Percentage body fat (%) | -4.159 | 0.578 | -0.275 | <0.001 | -5.294- -3.025 |
| Weekly physical activity (24-h MET) | 4.449 | 0.759 | 0.179 | <0.001 | 2.959-5.939 |
| Presence of inflammatory conditions | 17.795 | 7.509 | 0.071 | 0.018 | 3.057-32.534 |

*Linear regression analysis with GW/MFI-20 physical fatigue score as dependent variable. Sig.: p-value <0.05*

**Table A.5. Linear regression CPV- reduced activity ratio**

| **Variables** | **Unstandardized B** | **S.E.** | **Standardized B** | **Sig.** | **95% confidence interval** |
| --- | --- | --- | --- | --- | --- |
| **Log (10) hs-CRP** | -35.404 | 9.447 | -0.130 | <0.001 | -53.946- -16.862 |
| Cohort | 20.779 | 10.267 | 0.124 | 0.043 | 0.629-40.930 |
| Gender | -53.040 | 10.954 | -0.207 | <0.001 | 0.629-40.930 |
| Age (years) | -0.934 | 2.011 | -0.028 | 0.642 | -4.882-3.013 |
| Percentage body fat (%) | -2.112 | 0.679 | -0.135 | 0.002 | -3.445- -0.779 |
| Weekly physical activity (24-h MET) | 4.804 | 0.809 | 0.187 | <0.001 | 3.216-6.392 |
| Presence of inflammatory conditions | 8.883 | 8.035 | 0.034 | 0.269 | -6.886-24.653 |
|  |  |  |  |  |  |
| **Log (10) IL-6** | -53.445 | 12.046 | -0.146 | <0.001 | -77.088- -29.802 |
| Cohort | 21.683 | 10.204 | 0.129 | 0.034 | 1.656-41.709 |
| Gender | -51.567 | 10.644 | -0.202 | <0.001 | -72.457- -30.677 |
| Age (years) | -0.079 | 2.008 | -0.002 | 0.986 | -4.020-3.861 |
| Percentage body fat (%) | -2.305 | 0.633 | -0.148 | <0.001 | -3.548- -1.063 |
| Weekly physical activity (24-h MET) | 4.607 | 0.804 | 0.179 | <0.001 | 3.028-6.186 |
| Presence of inflammatory conditions | 9.394 | 7.980 | 0.037 | 0.239 | -6.268-25.056 |
|  |  |  |  |  |  |
| **Log (10) IL-10** | -14.801 | 6.578 | -0.070 | 0.025 | -27.711- -1.890 |
| Cohort | 21.101 | 10.320 | 0.126 | 0.041 | 0.846-41.355 |
| Gender | -42.508 | 10.534 | -0.166 | <0.001 | -63.184- -21.832 |
| Age (years) | -0.714 | 2.021 | -0.021 | 0.724 | -4.680-3.252 |
| Percentage body fat (%) | -3.114 | 0.617 | -0.199 | <0.001 | -4.324- -1.903 |
| Weekly physical activity (24-h MET) | 4.743 | 0.813 | 0.185 | <0.001 | 3.147-6.339 |
| Presence of inflammatory conditions | 11.056 | 8.061 | 0.043 | 0.171 | -4.766-26.878 |
|  |  |  |  |  |  |
| **Log (10) IFN-γ** | -13.591 | 10.411 | -0.041 | 0.192 | -34.024-6.842 |
| Cohort | 22.104 | 10.326 | 0.132 | 0.033 | 1.837-42.370 |
| Gender | -43.216 | 10.551 | -0.169 | <0.001 | -63.924- -22.509 |
| Age (years) | -0.777 | 2.022 | -0.023 | 0.701 | -4.746-3.192 |
| Percentage body fat (%) | -3.009 | 0.617 | -0.193 | <0.001 | -4.220- -1.798 |
| Weekly physical activity (24-h MET) | 4.777 | 0.813 | 0.186 | <0.001 | 3.181-6.372 |
| Presence of inflammatory conditions | 13.584 | 8.045 | 0.053 | 0.092 | -2.206-29.375 |
|  |  |  |  |  |  |
| **Log (10) TNF-alpha** | -50.096 | 21.520 | -0.074 | 0.020 | -92.333- -7.859 |
| Cohort | 22.885 | 10.305 | 0.137 | 0.027 | 2.660-43.110 |
| Gender | -46.326 | 10.643 | -0.181 | <0.001 | -67.215- -25.437 |
| Age (years) | -0.423 | 2.026 | -0.012 | 0.835 | -4.399-3.553 |
| Percentage body fat (%) | -2.929 | 0.617 | -0.188 | <0.001 | -4.140- -1.719 |
| Weekly physical activity (24-h MET) | 4.676 | 0.810 | 0.182 | <0.001 | 3.087-6.266 |
| Presence of inflammatory conditions | 10.020 | 8.012 | 0.047 | 0.134 | -3.705-27.744 |

*Linear regression analysis with GW/MFI-20 reduced activity scoreas dependent variable. Sig.: p-value <0.05*

**Table A.6. Linear regression on CPV- reduced motivation ratio**

| **Variables** | **Unstandardized B** | **S.E.** | **Standardized B** | **Sig.** | **95% confidence interval** |
| --- | --- | --- | --- | --- | --- |
| **Log (10) hs-CRP** | -30.091 | 9.354 | -0.114 | 0.001 | -48.450- -11.732 |
| Cohort | 16.391 | 10.166 | 0.101 | 0.107 | -3.561-36.343 |
| Gender | -60.549 | 10.846 | -0.246 | <0.001 | -81.836- -39.262 |
| Age (years) | -0.067 | 1.992 | -0.002 | 0.973 | -3.975-3.842 |
| Percentage body fat (%) | -0.792 | 0.672 | -0.052 | 0.239 | -2.112-0.528 |
| Weekly physical activity (24-h MET) | 3.990 | 0.801 | 0.161 | <0.001 | 2.418-5.562 |
| Presence of inflammatory conditions | 10.481 | 7.956 | 0.042 | 0.188 | -5.133-26.096 |
|  |  |  |  |  |  |
| **Log (10) IL-6** | -39.209 | 11.980 | -0.111 | 0.001 | -62.723- -15.695 |
| Cohort | 17.085 | 10.148 | 0.106 | 0.093 | -2.832-37.002 |
| Gender | -57.864 | 10.585 | -0.235 | <0.001 | -78.640- -37.089 |
| Age (years) | 0.560 | 1.997 | 0.017 | 0.779 | -3.358-4.479 |
| Percentage body fat (%) | -1.059 | 0.629 | -0.070 | 0.093 | -2.295-0.176 |
| Weekly physical activity (24-h MET) | 3.843 | 0.800 | 0.155 | <0.001 | 2.273-5.413 |
| Presence of inflammatory conditions | 11.112 | 7.936 | 0.045 | 0.162 | -4.464-26.688 |
|  |  |  |  |  |  |
| **Log (10) IL-10** | -15.724 | 6.506 | -0.078 | 0.016 | -28.494- -2.954 |
| Cohort | 16.733 | 10.207 | 0.103 | 0.102 | -3.301-36.767 |
| Gender | -51.345 | 10.420 | -0.208 | <0.001 | -71.795- -30.894 |
| Age (years) | 0.157 | 1.999 | 0.005 | 0.937 | -3.766-4.080 |
| Percentage body fat (%) | -1.678 | 0.610 | -0.111 | 0.006 | -2.875- -0.481 |
| Weekly physical activity (24-h MET) | 3.936 | 0.804 | 0.153 | <0.001 | 2.357-5.514 |
| Presence of inflammatory conditions | 11.757 | 7.974 | 0.047 | 0.141 | -3.893-27.407 |
|  |  |  |  |  |  |
| **Log (10) IFN-γ** | -18.540 | 10.285 | -0.058 | 0.072 | -38.728-1.647 |
| Cohort | 18.067 | 10.201 | 0.112 | 0.077 | -1.955-38.090 |
| Gender | -52.386 | 10.424 | -0.213 | <0.001 | -72.844- -31.927 |
| Age (years) | 0.135 | 1.998 | 0.004 | 0.946 | -3.786-4.056 |
| Percentage body fat (%) | -1.524 | 0.609 | 0.162 | 0.013 | 2.427-5.579 |
| Weekly physical activity (24-h MET) | 4.003 | 0.803 | 0.162 | <0.001 | 2.427-5.579 |
| Presence of inflammatory conditions | 14.845 | 7.948 | 0.060 | 0.062 | -0.755-30.445 |
|  |  |  |  |  |  |
| **Log (10) TNF-alpha** | -43.750 | 21.309 | -0.067 | 0.040 | -85.573 - -1.928 |
| Cohort | 18.184 | 10.204 | 0.112 | 0.075 | -1.843 - 38.211 |
| Gender | -54.627 | 10.539 | -0.222 | <0.001 | -75.312 - -33.943 |
| Age (years) | 0.369 | 2.006 | 0.011 | 0.854 | -3.568 - 4.306 |
| Percentage body fat (%) | -1.498 | 0.611 | -0.099 | 0.014 | -2.697 - -0.299 |
| Weekly physical activity (24-h MET) | 3.883 | 0.802 | 0.157 | <0.001 | 2.309 - 5.457 |
| Presence of inflammatory conditions | 12.991 | 7.933 | 0.052 | 0.102 | -2.580 - 25.561 |

*Linear regression analysis with GW/MFI-20 reduced motivation score as dependent variable. Sig.: p-value <0.05*

**Table A.7. Linear regression on CPV- mental fatigue ratio**

| **Variables** | **Unstandardized B** | **S.E.** | **Standardized B** | **Sig.** | **95% confidence interval** |
| --- | --- | --- | --- | --- | --- |
| **Log (10) hs-CRP** | -26.913 | 10.176 | -0.095 | 0.008 | -46.885 - -6.940 |
| Cohort | 26.260 | 11.099 | 0.150 | 0.018 | 4.477 - 48.043 |
| Gender | -84.900 | 11.800 | -0.319 | <0.001 | -108.060 - -61.740 |
| Age (years) | 0.978 | 2.184 | 0.028 | 0.654 | -3.308 - 5.264 |
| Percentage body fat (%) | 0.466 | 0.732 | 0.029 | 0.525 | -0.970 - 1.901 |
| Weekly physical activity (24-h MET) | 2.957 | 0.871 | 0.111 | 0.001 | 1.247 - 4.667 |
| Presence of inflammatory conditions | 21.121 | 8.656 | 0.079 | 0.015 | 4.132 - 38.110 |
|  |  |  |  |  |  |
| **Log (10) IL-6** | -38.724 | 12.980 | -0.102 | 0.003 | -64.201 - -13.248 |
| Cohort | 26.730 | 11.036 | 0.154 | 0.016 | 5.070 - 48.390 |
| Gender | -83.658 | 11.471 | -0.315 | <0.001 | -106.173 - -61.144 |
| Age (years) | 1.594 | 2.180 | 0.045 | 0.465 | -2.686 - 5.873 |
| Percentage body fat (%) | 0.321 | 0.682 | 0.020 | 0.638 | -1.018 - 1.659 |
| Weekly physical activity (24-h MET) | 2.834 | 0.867 | 0.106 | 0.001 | 1.133 - 4.536 |
| Presence of inflammatory conditions | 21.447 | 8.601 | 0.080 | 0.013 | 4.566 - 38.329 |
|  |  |  |  |  |  |
| **Log (10) IL-10** | -16.456 | 7.045 | -0.075 | 0.020 | -30.283 - -2.628 |
| Cohort | 26.481 | 11.090 | 0.152 | 0.017 | 4.714 - 48.248 |
| Gender | -77.261 | 11.282 | -0.291 | <0.001 | -99.404 - -55.119 |
| Age (years) | 1.194 | 2.181 | 0.034 | 0.584 | -3.086 - 5.475 |
| Percentage body fat (%) | -0.301 | 0.660 | -0.018 | 0.649 | -1.597 - 0.996 |
| Weekly physical activity (24-h MET) | 2.928 | 0.871 | 0.110 | 0.001 | 1.219 - 4.637 |
| Presence of inflammatory conditions | 21.979 | 8.634 | 0.082 | 0.011 | 5.033 - 38.925 |
|  |  |  |  |  |  |
| **Log (10) IFN-γ** | -18.664 | 11.151 | -0.054 | 0.095 | -40.550 - 3.222 |
| Cohort | 27.394 | 11.101 | 0.157 | 0.014 | 5.606 - 49.181 |
| Gender | -78.295 | 11.302 | -0.295 | <0.001 | -100.478 - -56.113 |
| Age (years) | 1.147 | 2.183 | 0.032 | 0.600 | -3.139 - 5.432 |
| Percentage body fat (%) | -0.158 | 0.661 | -0.010 | 0.812 | -1.455 - 1.140 |
| Weekly physical activity (24-h MET) | 2.972 | 0.871 | 0.111 | 0.001 | 1.263 - 4.681 |
| Presence of inflammatory conditions | 25.149 | 8.620 | 0.094 | 0.004 | 8.231 - 42.066 |
|  |  |  |  |  |  |
| **Log (10) TNF-alpha** | -37.010 | 23.100 | -0.053 | 0.109 | -82.348 - 8.328 |
| Cohort | 27.623 | 11.101 | 0.159 | 0.013 | 5.836 - 49.411 |
| Gender | -80.148 | 11.426 | -0.302 | <0.001 | -102.575 - -57.722 |
| Age (years) | 1.319 | 2.191 | 0.037 | 0.547 | -2.982 - 5.619 |
| Percentage body fat (%) | -0.134 | 0.662 | -0.008 | 0.839 | -1.434 - 1.165 |
| Weekly physical activity (24-h MET) | 2.862 | 0.869 | 0.107 | 0.001 | 1.155 - 4.568 |
| Presence of inflammatory conditions | 23.567 | 8.602 | 0.088 | 0.006 | 6.684 - 40.451 |

*Linear regression analysis with GW/MFI-20 mental fatigue score as dependent variable. Sig.: p-value <0.05*

**Table A.8. Linear regression on CPV- total ratio for per persons with inflammatory conditions**

| **Variables** | **Unstandardized B** | **S.E.** | **Standardized B** | **Sig.** | **95% confidence interval** |
| --- | --- | --- | --- | --- | --- |
| **Log (10) hs-CRP** | -5.281 | 1.869 | -0.124 | 0.005 | -8.954- -1.609 |
| Cohort | 4.604 | 2.130 | 0.175 | 0.031 | 0.419-8.788 |
| Gender | -10.785 | 2.285 | -0.257 | <0.001 | -15.275- -6.296 |
| Age (years) | 0.114 | 0.425 | 0.021 | 0.790 | -0.722-0.949 |
| Percentage body fat (%) | -0.372 | 0.136 | -0.151 | 0.007 | -0.639- -0.104 |
| Weekly physical activity (24-h MET) | 0.707 | 0.159 | 0.183 | <0.001 | 0.394-1.020 |
|  |  |  |  |  |  |
| **Log (10) IL-6** | -7.022 | 2.407 | -0.123 | 0.004 | -12.752- -2.293 |
| Cohort | 4.785 | 2.118 | 0.182 | 0.024 | 0.624-8.945 |
| Gender | -10.840 | 2.252 | -0.259 | <0.001 | -15.265- -6.415 |
| Age (years) | 0.221 | 0.424 | 0.041 | 0.602 | -0.612-1.054 |
| Percentage body fat (%) | -0.395 | 0.128 | -0.162 | 0.002 | -0.647- -0.143 |
| Weekly physical activity (24-h MET) | 0.689 | 0.159 | 0.179 | <0.001 | 0.377-1.001 |
|  |  |  |  |  |  |
| **Log (10) IL-10** | -2.418 | 1.331 | -0.073 | 0.070 | -5.033- -0.197 |
| Cohort | 4.685 | 2.140 | 0.178 | 0.029 | 0.480-8.890 |
| Gender | -9.586 | 2.217 | -0.229 | <0.001 | -13.942- -5.230 |
| Age (years) | 0.126 | 0.427 | 0.023 | 0.768 | -0.712-0.964 |
| Percentage body fat (%) | -0.503 | 0.125 | -0.205 | <0.001 | -0.748- -0.258 |
| Weekly physical activity (24-h MET) | 0.714 | 0.160 | 0.185 | <0.001 | 0.400-1.029 |
|  |  |  |  |  |  |
| **Log (10) IFN-γ** | -0.518 | 2.161 | -0.010 | 0.811 | -4.764- 3.728 |
| Cohort | 4.894 | 2.140 | 0.186 | 0.023 | 0.690-9.098 |
| Gender | -9.447 | 2.217 | -0.226 | <0.001 | -13.802- -5.092 |
| Age (years) | 0.143 | 0.427 | 0.026 | 0.738 | -0.696-0.981 |
| Percentage body fat (%) | -0.490 | 0.125 | -0.201 | <0.001 | -0.735- -0.245 |
| Weekly physical activity (24-h MET) | 0.715 | 0.160 | 0.185 | <0.001 | 0.400-1.029 |
|  |  |  |  |  |  |
| **Log (10) TNF-alpha** | -4.885 | 4.404 | -0.046 | 0.268 | -13.538- 3.768 |
| Cohort | 4.858 | 2.133 | 0.185 | 0.023 | 0.666-9.049 |
| Gender | -9.852 | 2.251 | -0.235 | <0.001 | -14.275- -5.430 |
| Age (years) | 0.174 | 0.427 | 0.032 | 0.684 | -0.665-1.013 |
| Percentage body fat (%) | -0.479 | 0.125 | -0.196 | <0.001 | -0.725- -0.233 |
| Weekly physical activity (24-h MET) | 0.707 | 0.160 | 0.183 | <0.001 | 0.393-1.021 |

*Linear regression analysis with GW/MFI-20 total fatigue score as dependent variable. Sig.: p-value <0.05*

**Table A.9. Linear regression on CPV- total ratio for per persons without inflammatory conditions**

| **Variables** | **Unstandardized B** | **S.E.** | **Standardized B** | **Sig.** | **95% confidence interval** |
| --- | --- | --- | --- | --- | --- |
| **Log (10) hs-CRP** | -7.205 | 2.719 | -0.144 | 0.008 | -12.552- -1.859 |
| Cohort | 4.801 | 2.744 | 0.161 | 0.081 | -0.595-10.197 |
| Gender | -14.817 | 2.911 | -0.351 | <0.001 | -20.540- -9.093 |
| Age (years) | 0.213 | 0.521 | 0.037 | 0.683 | -0.811-1.238 |
| Percentage body fat (%) | -0.003 | 0.192 | -0.001 | 0.988 | -0.380- -0.375 |
| Weekly physical activity (24-h MET) | 0.623 | 0.239 | 0.127 | 0.009 | 0.153-1.092 |
|  |  |  |  |  |  |
| **Log (10) IL-6** | -12.416 | 3.410 | -0.186 | <0.001 | -19.122- -5.711 |
| Cohort | 4.880 | 2.722 | 0.163 | 0.074 | -0.473-10.234 |
| Gender | -13.831 | 2.763 | -0.328 | <0.001 | -19.265- -8.398 |
| Age (years) | 0.423 | 0.521 | 0.073 | 0.417 | -0.602-1.488 |
| Percentage body fat (%) | -0.059 | 0.176 | -0.021 | 0.737 | -0.406- -0.287 |
| Weekly physical activity (24-h MET) | 0.567 | 0.236 | 0.115 | 0.017 | 0.102-1.032 |
|  |  |  |  |  |  |
| **Log (10) IL-10** | -2.282 | 1.840 | -0.062 | 0.216 | -5.901- -1.337 |
| Cohort | 4.759 | 2.767 | 0.159 | 0.086 | -0.681-10.199 |
| Gender | -12.027 | 2.768 | -0.285 | <0.001 | -17.470- -6.584 |
| Age (years) | 0.257 | 0.529 | 0.044 | 0.628 | -0.784-1.297 |
| Percentage body fat (%) | -0.245 | 0.173 | -0.089 | 0.156 | -0.585- -0.094 |
| Weekly physical activity (24-h MET) | 0.574 | 0.240 | 0.117 | 0.017 | 0.101-1.046 |
|  |  |  |  |  |  |
| **Log (10) IFN-γ** | -7.658 | 2.777 | -0.135 | 0.006 | -13.118- -2.197 |
| Cohort | 5.758 | 2.771 | 0.193 | 0.038 | 0.309-11.208 |
| Gender | -13.004 | 2.755 | -0.308 | <0.001 | -18.422- -7.587 |
| Age (years) | 0.376 | 0.526 | 0.064 | 0.476 | -0.659-1.410 |
| Percentage body fat (%) | -0.201 | 0.171 | -0.073 | 0.241 | -0.538- 0.136 |
| Weekly physical activity (24-h MET) | 0.610 | 0.238 | 0.124 | 0.011 | 0.142-1.079 |
|  |  |  |  |  |  |
| **Log (10) TNF-alpha** | -9.916 | 5.901 | -0.083 | 0.094 | -21.519- 1.687 |
| Cohort | 5.248 | 2.783 | 0.176 | 0.060 | -0.224-10.720 |
| Gender | -12.803 | 2.779 | -0.303 | <0.001 | -18.268- -7.339 |
| Age (years) | 0.301 | 0.530 | 0.052 | 0.571 | -0.742-1.343 |
| Percentage body fat (%) | -0.218 | 0.172 | -0.079 | 0.206 | -0.557- 0.120 |
| Weekly physical activity (24-h MET) | 0.569 | 0.239 | 0.116 | 0.018 | 0.099-1.040 |

*Linear regression analysis with GW/MFI-20 total fatigue score as dependent variable. Sig.: p-value <0.05*

**Table A.10. Linear regression on CPV- general ratio for per persons with inflammatory conditions**

| **Variables** | **Unstandardized B** | **S.E.** | **Standardized B** | **Sig.** | **95% confidence interval** |
| --- | --- | --- | --- | --- | --- |
| **Log (10) hs-CRP** | -21.408 | 9.801 | -0.099 | 0.029 | -40.644- -2.153 |
| Cohort | 27.325 | 2.130 | 0.203 | 0.015 | 5.383-49.267 |
| Gender | -64.460 | 2.285 | -0.301 | <0.001 | -88.002- -40.919 |
| Age (years) | 1.070 | 0.425 | 0.039 | 0.632 | -3.312-5.453 |
| Percentage body fat (%) | -1.126 | 0.136 | -0.090 | 0.115 | -2.529- -0.277 |
| Weekly physical activity (24-h MET) | 2.637 | 0.159 | 0.133 | 0.002 | 0.996-4.278 |
|  |  |  |  |  |  |
| **Log (10) IL-6** | -38.776 | 12.541 | -0.134 | 0.002 | -63.416- -14.135 |
| Cohort | 28.183 | 11.032 | 0.211 | 0.011 | 6.508-49.859 |
| Gender | -67.436 | 11.735 | -0.317 | <0.001 | -90.619- -44.508 |
| Age (years) | 1.663 | 2.208 | 0.060 | 0.452 | -2.676-6.002 |
| Percentage body fat (%) | -1.029 | 0.667 | -0.083 | 0.124 | -2.340- 0.283 |
| Weekly physical activity (24-h MET) | 2.534 | 0.826 | 0.129 | 0.002 | 0.911-4.157 |
|  |  |  |  |  |  |
| **Log (10) IL-10** | -15.551 | 6.928 | -0.092 | 0.025 | -29.163- -1.939 |
| Cohort | 27.623 | 11.140 | 0.206 | 0.013 | 5.736-49.509 |
| Gender | -60.813 | 11.539 | -0.284 | <0.001 | -83.485- -5.484 |
| Age (years) | 1.122 | 2.220 | 0.041 | 0.613 | -3.239-5.484 |
| Percentage body fat (%) | -1.636 | 0.649 | -0.131 | 0.012 | -2.910- -0.361 |
| Weekly physical activity (24-h MET) | 2.678 | 0.834 | 0.136 | 0.001 | 1.040-4.316 |
|  |  |  |  |  |  |
| **Log (10) IFN-γ** | -2.749 | 11.275 | -0.010 | 0.807 | -24.901- 19.403 |
| Cohort | 28.645 | 11.164 | 0.214 | 0.011 | 6.710-50.597 |
| Gender | -59.818 | 11.564 | -0.280 | <0.001 | -82.539- -37.097 |
| Age (years) | 1.225 | 2.226 | 0.044 | 0.582 | -3.149-5.599 |
| Percentage body fat (%) | -1.563 | 0.650 | -0.126 | 0.017 | -2.840- -0.286 |
| Weekly physical activity (24-h MET) | 2.672 | 0.835 | 0.136 | <0.001 | 1.032-4.132 |
|  |  |  |  |  |  |
| **Log (10) TNF-alpha** | -9.923 | 22.992 | -0.018 | 0.666 | -55.096-35.251 |
| Cohort | 28.395 | 11.138 | 0.212 | 0.011 | 6.512-50.279 |
| Gender | -60.526 | 11.752 | -0.284 | <0.001 | -83.615- -37.437 |
| Age (years) | 1.285 | 2.230 | 0.047 | 0.565 | -3.096-5.666 |
| Percentage body fat (%) | -1.559 | 0.654 | -0.125 | 0.017 | -2.844- -0.275 |
| Weekly physical activity (24-h MET) | 2.646 | 0.833 | 0.135 | 0.002 | 1.009-4.283 |

*Linear regression analysis with GW/MFI-20 general fatigue score as dependent variable. Sig.: p-value <0.05*

**Table A.11. Linear regression on CPV- general ratio for per persons without inflammatory conditions**

| **Variables** | **Unstandardized B** | **S.E.** | **Standardized B** | **Sig.** | **95% confidence interval** |
| --- | --- | --- | --- | --- | --- |
| **Log (10) hs-CRP** | -29.968 | 15.363 | -0.108 | 0.052 | -60.178- 0.242 |
| Cohort | 32.538 | 15.505 | 0.195 | 0.037 | 2.049-63.026 |
| Gender | -83.448 | 16.446 | -0.354 | <0.001 | -115.789- -51.107 |
| Age (years) | 2.284 | 2.944 | 0.070 | 0.438 | -3.505-8.073 |
| Percentage body fat (%) | 0.368 | 1.085 | 0.024 | 0.735 | -1.765- 2.500 |
| Weekly physical activity (24-h MET) | 2.640 | 1.348 | 0.096 | 0.051 | -0.011-5.291 |
|  |  |  |  |  |  |
| **Log (10) IL-6** | -54.402 | 19.340 | -0.146 | 0.005 | -92.432- -16.371 |
| Cohort | 32.950 | 15.441 | 0.198 | 0.034 | 2.586-63.314 |
| Gender | -80.177 | 15.672 | -0.340 | <0.001 | -110.996- -49.358 |
| Age (years) | 3.154 | 2.956 | 0.097 | 0.287 | -2.660-8.697 |
| Percentage body fat (%) | 0.162 | 0.999 | 0.010 | 0.871 | -1.803- 2.127 |
| Weekly physical activity (24-h MET) | 2.352 | 1.341 | 0.086 | 1.754 | -0.285-4.990 |
|  |  |  |  |  |  |
| **Log (10) IL-10** | -2.344 | 10.385 | -0.011 | 0.822 | -22.767- 18.078 |
| Cohort | 31.979 | 15.612 | 0.192 | 0.041 | -1.279-62.679 |
| Gender | -72.637 | 15.621 | -0.308 | <0.001 | -103.355- -41.920 |
| Age (years) | 2.147 | 2.989 | 0.066 | 0.473 | -3.725-8.019 |
| Percentage body fat (%) | -0.606 | 0.975 | -0.039 | 0.534 | -2.524- 1.311 |
| Weekly physical activity (24-h MET) | 2.424 | 1.356 | 0.088 | 0.075 | -0.243-5.090 |
|  |  |  |  |  |  |
| **Log (10) IFN-γ** | -42.931 | 15.625 | -0.136 | 0.006 | -73.657- -12.206 |
| Cohort | 38.149 | 15.593 | 0.229 | 0.015 | 7.486-68.811 |
| Gender | -77.022 | 15.501 | -0.327 | <0.001 | -107.503- -46.540 |
| Age (years) | 3.272 | 2.960 | 0.101 | 0.270 | -2.548-9.092 |
| Percentage body fat (%) | -0.411 | 0.964 | -0.027 | 0.670 | -2.307- 1.485 |
| Weekly physical activity (24-h MET) | 2.641 | 1.340 | 0.096 | 0.049 | 0.007-5.275 |
|  |  |  |  |  |  |
| **Log (10) TNF-alpha** | -71.792 | 33.114 | -0.108 | 0.031 | -136.910- -6.675 |
| Cohort | 36.292 | 15.617 | 0.218 | 0.021 | 5.581-67.002 |
| Gender | -76.820 | 15.596 | -0.326 | <0.001 | -107.488- -46.152 |
| Age (years) | 3.081 | 2.975 | 0.095 | 0.301 | -2.770-8.932 |
| Percentage body fat (%) | -0.484 | 0.967 | -0.031 | 0.617 | -2.385- 1.418 |
| Weekly physical activity (24-h MET) | 2.403 | 1.343 | 0.088 | 0.074 | -0.238-5.043 |

*Linear regression analysis with GW/MFI-20 general fatigue score as dependent variable. Sig.: p-value <0.05*

**Table A.12. Linear regression on CPV- physical ratio for per persons with inflammatory conditions**

| **Variables** | **Unstandardized B** | **S.E.** | **Standardized B** | **Sig.** | **95% confidence interval** |
| --- | --- | --- | --- | --- | --- |
| **Log (10) hs-CRP** | -30.703 | 10.829 | -0.121 | 0.005 | -51.987- -9.427 |
| Cohort | 37.167 | 12.340 | 0.237 | 0.003 | 12.923-61.411 |
| Gender | -43.239 | 13.239 | -0.173 | 0.001 | -69.250- -17.229 |
| Age (years) | 2.497 | 2.465 | 0.077 | 0.311 | -2.345-7.340 |
| Percentage body fat (%) | -3.602 | 7.89 | -0.246 | <0.001 | -5.152- -2.052 |
| Weekly physical activity (24-h MET) | 4.976 | 0.923 | 0.216 | <0.001 | 3.163 – 6.789 |
|  |  |  |  |  |  |
| **Log (10) IL-6** | -47.790 | 13.992 | -0.140 | <0.001 | -75.280- -20.299 |
| Cohort | 38.039 | 12.308 | 0.242 | 0.002 | 13.857-62.222 |
| Gender | -43.276 | 13.092 | -0.173 | 0.001 | -68.999- -17.554 |
| Age (years) | 3.155 | 2.464 | 0.098 | 0.201 | -1.686- 7.996 |
| Percentage body fat (%) | -3.722 | 0.745 | -0.255 | <0.001 | -5.185- -2.259 |
| Weekly physical activity (24-h MET) | 4.846 | 0.922 | 0.210 | <0.001 | 3.035-6.657 |
|  |  |  |  |  |  |
| **Log (10) IL-10** | -4.799 | 7.781 | -0.024 | 0.583 | -20.087- 10.488 |
| Cohort | 38.138 | 12.511 | 0.242 | 0.002 | 13.557-62.719 |
| Gender | -33.949 | 12.959 | -0.135 | 0.009 | -59.411- -8.487 |
| Age (years) | 2.606 | 2.493 | 0.080 | 0.296 | -2.292- 7.505 |
| Percentage body fat (%) | -4.469 | 0.729 | -0.305 | <0.001 | -5.901- -3.038 |
| Weekly physical activity (24-h MET) | 4.967 | 0.963 | 0.215 | <0.001 | 3.127-6.806 |
|  |  |  |  |  |  |
| **Log (10) IFN-γ** | -6.480 | 12.594 | -0.020 | 0.607 | -31.224- 18.265 |
| Cohort | 38.633 | 12.471 | 0.245 | 0.002 | 14.132- 63.135 |
| Gender | -33.832 | 12.917 | -0.135 | 0.009 | -59.211- -8.452 |
| Age (years) | 2.606 | 2.487 | 0.081 | 0.295 | -2.280- 7.492 |
| Percentage body fat (%) | -4.361 | 0.726 | -0.299 | <0.001 | -5.788- -2.935 |
| Weekly physical activity (24-h MET) | 5.031 | 0.932 | 0.218 | <0.001 | 3.199- 6.863 |
|  |  |  |  |  |  |
| **Log (10) TNF-alpha** | -21.767 | 25.691 | -0.034 | 0.397 | -72.242- 28.708 |
| Cohort | 38.407 | 12.446 | 0.245 | 0.002 | 13.955- 62.859 |
| Gender | -35.489 | 13.131 | -0.142 | 0.007 | -61.287- -9.690 |
| Age (years) | 2.754 | 2.492 | 0.082 | 0.270 | -2.141- 7.650 |
| Percentage body fat (%) | -4.340 | 0.730 | -0.297 | <0.001 | -5.775- -2.905 |
| Weekly physical activity (24-h MET) | 4.977 | 0.931 | 0.216 | <0.001 | 3.148- 6.806 |

*Linear regression analysis with GW/MFI-20 physical fatigue score as dependent variable. Sig.: p-value <0.05*

**Table A.13. Linear regression on CPV- physical ratio for per persons without inflammatory conditions**

| **Variables** | **Unstandardized B** | **S.E.** | **Standardized B** | **Sig.** | **95% confidence interval** |
| --- | --- | --- | --- | --- | --- |
| **Log (10) hs-CRP** | -40.706 | 15.142 | -0.142 | 0.008 | -70.481- -10.931 |
| Cohort | 29.467 | 15.281 | 0.172 | 0.055 | -0.582 – 59.517 |
| Gender | -66.784 | 16.21 | -0.275 | <0.001 | -98.659- -34.909 |
| Age (years) | 0.586 | 2.902 | 0.018 | 0.840 | -5.119- 6.292 |
| Percentage body fat (%) | -2.546 | 1.069 | -0.160 | 0.018 | -4.648- -0.444 |
| Weekly physical activity (24-h MET) | 3.663 | 1.329 | 0.130 | 0.006 | 1.050-6.275 |
|  |  |  |  |  |  |
| **Log (10) IL-6** | -58.209 | 19.095 | -0.151 | 0.002 | -95.758- -2.0660 |
| Cohort | 29.673 | 15.246 | 0.173 | 0.052 | -0.307- 59.652 |
| Gender | -59.600 | 15.474 | -0.246 | <0.001 | -90.029- -29.170 |
| Age (years) | 1.533 | 2.919 | 0.046 | 0.600 | -4.207-7.273 |
| Percentage body fat (%) | -3.031 | 9.87 | -0.191 | 0.002 | -4.972- -1.090 |
| Weekly physical activity (24-h MET) | 3.365 | 1.324 | 0.119 | 0.011 | 0.761-5.969 |
|  |  |  |  |  |  |
| **Log (10) IL-10** | -7.510 | 10.266 | -0.035 | 0.465 | -27.698- 12.678 |
| Cohort | 28.922 | 15.433 | 0.169 | 0.062 | -1.425- 59.269 |
| Gender | -51.293 | 15.441 | -0.212 | <0.001 | -81.657- -20.928 |
| Age (years) | 0.637 | 2.952 | 0.019 | 0.829 | -5.167- 6.441 |
| Percentage body fat (%) | -3.884 | 0.964 | -0.244 | <0.001 | -5.779- -1.988 |
| Weekly physical activity (24-h MET) | 3.415 | 1.341 | 0.121 | 0.011 | 0.779-6.051 |
|  |  |  |  |  |  |
| **Log (10) IFN-γ** | -42.178 | 15.473 | -0.130 | 0.007 | -72.605- -11.752 |
| Cohort | 34.718 | 15.441 | 0.202 | 0.025 | 4.354- 62.082 |
| Gender | -56.437 | 15.350 | -0.233 | <0.001 | -86.622- -26.251 |
| Age (years) | 1.475 | 2.931 | 0.044 | 0.615 | -4.289- 7.239 |
| Percentage body fat (%) | -3.672 | 0.955 | -0.231 | <0.001 | -5.549- -1.794 |
| Weekly physical activity (24-h MET) | 3.589 | 1.326 | 0.127 | 0.007 | 0.980- 6.197 |
|  |  |  |  |  |  |
| **Log (10) TNF-alpha** | -37.795 | 32.937 | -0.055 | 0.525 | -102.56-26.974 |
| Cohort | 30.864 | 15.534 | 0.180 | 0.048 | 0.318-61.410 |
| Gender | -54.367 | 15.512 | -0.224 | <0.001 | -84.871—23.863 |
| Age (years) | 0.825 | 2.959 | 0.025 | 0.781 | -4.995-6.644 |
| Percentage body fat (%) | -3.792 | 0.962 | -0.238 | <0.001 | -5.683- -1.901 |
| Weekly physical activity (24-h MET) | 3.372 | 1.336 | 0.119 | 0.012 | 0.745-5.998 |

*Linear regression analysis with GW/MFI-20 physical fatigue score as dependent variable. Sig.: p-value <0.05*

**Table A.14. Linear regression on CPV- reduced activation ratio for per persons with inflammatory conditions**

| **Variables** | **Unstandardized B** | **S.E.** | **Standardized B** | **Sig.** | **95% confidence interval** |
| --- | --- | --- | --- | --- | --- |
| **Log (10) hs-CRP** | -34.021 | 11.799 | -0.128 | 0.004 | -57.202- -10.839 |
| Cohort | 17.951 | 13.445 | 0.109 | 0.182 | -8.465- 44.366 |
| Gender | -42.349 | 14.425 | -0.161 | 0.003 | -70.690- -14.008 |
| Age (years) | -1.889 | 2.686 | -0.056 | 0.482 | -7.166- 3.387 |
| Percentage body fat (%) | -2.849 | 0.860 | -0.185 | <0.001 | -4.538 - -1.161 |
| Weekly physical activity (24-h MET) | 4.979 | 1.005 | 0.205 | <0.001 | 3.003- 6.954 |
|  |  |  |  |  |  |
| **Log (10) IL-6** | -37.077 | 15.237 | -0.104 | 0.015 | -67.015- -7.140 |
| Cohort | 19.417 | 13.404 | 0.118 | 0.148 | -6.918- 45.753 |
| Gender | -41.102 | 14.258 | -0.157 | 0.004 | -69.114- -13.090 |
| Age (years) | -1.226 | 2.683 | -0.036 | 0.648 | -6.497- 4.046 |
| Percentage body fat (%) | -3.123 | 0.811 | -0.204 | <0.001 | -4.716- -1.530 |
| Weekly physical activity (24-h MET) | 4.879 | 1.004 | 0.202 | <0.001 | 2.907-6.852 |
|  |  |  |  |  |  |
| **Log (10) IL-10** | -14.866 | 8.406 | -0.072 | 0.078 | -31.381- 1.649 |
| Cohort | 18.467 | 13.515 | 0.112 | 0.172 | -8.087- 45.021 |
| Gender | -35.578 | 14.000 | -0.132 | 0.014 | -62.084- -7.072 |
| Age (years) | -1.753 | 2.693 | -0.052 | 0.515 | -7.045- 3.538 |
| Percentage body fat (%) | -3.674 | 0.787 | -0.239 | <0.001 | -5.220- -2.128 |
| Weekly physical activity (24-h MET) | 5.034 | 1.011 | 0.208 | <0.001 | 3.047- 7.021 |
|  |  |  |  |  |  |
| **Log (10) IFN-γ** | 2.227 | 13.655 | 0.007 | 0.871 | -24.601- 29.055 |
| Cohort | 20.003 | 13.521 | 0.121 | 0.140 | -6.561- 46.568 |
| Gender | -33.605 | 14.005 | -0.128 | 0.017 | -61.122- -6.089 |
| Age (years) | -1.623 | 2.696 | -0.148 | 0.548 | -6.920- 3.674 |
| Percentage body fat (%) | -3.656 | 0.787 | -0.239 | <0.001 | -5.203- -2.109 |
| Weekly physical activity (24-h MET) | 4.991 | 1.011 | 0.206 | <0.001 | 3.005- 6.978 |
|  |  |  |  |  |  |
| **Log (10) TNF-alpha** | -40.654 | 27.780 | -0.061 | 0.144 | -95.235- 13.927 |
| Cohort | 19.967 | 13.458 | 0.121 | 0.139 | -6.474- 46.409 |
| Gender | -37.267 | 14.199 | -0.142 | 0.009 | -65.164- -9.370 |
| Age (years) | -1.375 | 2.964 | -0.041 | 0.610 | -6.667- 3.919 |
| Percentage body fat (%) | -3.511 | 0.790 | -0.230 | <0.001 | -5.063- -1.960 |
| Weekly physical activity (24-h MET) | 4.963 | 1.007 | 0.205 | <0.001 | 2.985- 6.941 |

*Linear regression analysis with GW/MFI-20 reduced activation fatigue score as dependent variable. Sig.: p-value <0.05*

**Table A.15. Linear regression on CPV- reduced activation ratio for per persons without inflammatory conditions**

| **Variables** | **Unstandardized B** | **S.E.** | **Standardized B** | **Sig.** | **95% confidence interval** |
| --- | --- | --- | --- | --- | --- |
| **Log (10) hs-CRP** | -38.533 | 15.916 | -0.133 | 0.016 | -69.831- -7.236 |
| Cohort | 23.727 | 16.063 | 0.137 | 0.140 | -7.859- 55.313 |
| Gender | -70.123 | 17.038 | -0.286 | <0.001 | -103.628- -36.618 |
| Age (years) | 0.305 | 3.050 | 0.009 | 0.920 | -5.692- 6.302 |
| Percentage body fat (%) | -0.737 | 1.124 | -0.046 | 0.512 | -2.946- 1.473 |
| Weekly physical activity (24-h MET) | 4.076 | 1.397 | 0.143 | 0.004 | 1.330-6.823 |
|  |  |  |  |  |  |
| **Log (10) IL-6** | -82.812 | 19.824 | -0.213 | <0.001 | -121.795- -43.829 |
| Cohort | 24.485 | 15.828 | 0.141 | 0.123 | -6.640- 55.610 |
| Gender | -67.101 | 16.065 | -0.273 | <0.001 | -98.692- -35.510 |
| Age (years) | 1.757 | 3.031 | 0.052 | 0.562 | -4.202- 7.717 |
| Percentage body fat (%) | -0.811 | 1.024 | -0.050 | 0.429 | -2.826- 1.203 |
| Weekly physical activity (24-h MET) | 3.753 | 1.375 | 0.131 | 0.007 | 1.049-6.456 |
|  |  |  |  |  |  |
| **Log (10) IL-10** | -15.605 | 10.750 | -0.072 | 0.147 | -36.745- 5.534 |
| Cohort | 23.701 | 16.160 | 0.137 | 0.143 | -8.077-55.480 |
| Gender | -55.047 | 16.170 | -0.224 | <0.001 | -86.844- -23.251 |
| Age (years) | 0.661 | 3.091 | 0.020 | 0.831 | -5.416- 6.739 |
| Percentage body fat (%) | -2.056 | 1.009 | -0.128 | 0.042 | -4.041- -0.071 |
| Weekly physical activity (24-h MET) | 3.797 | 1.404 | 0.133 | 0.007 | 1.037-6.558 |
|  |  |  |  |  |  |
| **Log (10) IFN-γ** | -38.347 | 16.275 | -0.117 | 0.019 | -70.351- -6.342 |
| Cohort | 28.465 | 16.242 | 0.164 | 0.081 | -3.473- 60.404 |
| Gender | -60.170 | 16.146 | -0.245 | <0.001 | -91.920- -28.419 |
| Age (years) | 1.102 | 3.083 | 0.033 | 0.721 | -4.961- 7.164 |
| Percentage body fat (%) | -1.809 | 1.004 | -0.112 | 0.072 | -3.784- 0.166 |
| Weekly physical activity (24-h MET) | 3.999 | 1.395 | 0.140 | 0.004 | 1.256- 6.743 |
|  |  |  |  |  |  |
| **Log (10) TNF-alpha** | -61.793 | 34.470 | -0.089 | 0.074 | -129.57- 5.990 |
| Cohort | 26.662 | 16.256 | 0.153 | 0.102 | -5.305- 58.629 |
| Gender | -59.856 | 16.234 | -0.244 | <0.001 | -91.779- -27.933 |
| Age (years) | 0.898 | 3.097 | 0.027 | 0.772 | -5.192- 6.989 |
| Percentage body fat (%) | -1.877 | 1.006 | -0.117 | 0.063 | -3.856- 0.102 |
| Weekly physical activity (24-h MET) | 3.787 | 1.398 | 0.133 | 0.007 | 1.039- 6.536 |

*Linear regression analysis with GW/MFI-20 reduced activation fatigue score as dependent variable. Sig.: p-value <0.05*

**Table A.16. Linear regression on CPV- reduced motivation ratio for per persons with inflammatory conditions**

| **Variables** | **Unstandardized B** | **S.E.** | **Standardized B** | **Sig.** | **95% confidence interval** |
| --- | --- | --- | --- | --- | --- |
| **Log (10) hs-CRP** | -25.493 | 11.443 | -0.101 | 0.026 | -47.974- -3.011 |
| Cohort | 15.050 | 13.039 | 0.096 | 0.249 | -10.568- 40.668 |
| Gender | -54.046 | 13.898 | -0.217 | <0.001 | -81.531- -26.561 |
| Age (years) | -0.400 | 2.604 | -0.012 | 0.878 | -5.517- 4.717 |
| Percentage body fat (%) | -1.687 | 0.834 | -0.116 | 0.044 | -3.325- -0.049 |
| Weekly physical activity (24-h MET) | 4.436 | 0.975 | 0.193 | <0.001 | 2.520- 6351 |
|  |  |  |  |  |  |
| **Log (10) IL-6** | -32.361 | 14.780 | -0.096 | 0.029 | -61.400- -3.323 |
| Cohort | 16.127 | 13.001 | 0.103 | 0.215 | -9.417- 41.671 |
| Gender | -53.769 | 13.829 | -0.216 | <0.001 | -80.939- -26.598 |
| Age (years) | 0.140 | 2.603 | 0.004 | 0.957 | -4.974- 5.253 |
| Percentage body fat (%) | -1.824 | 0.787 | -0.126 | 0.021 | -3.369- -0.279 |
| Weekly physical activity (24-h MET) | 4.360 | 0.974 | 0.190 | <0.001 | 2.447- 6.227 |
|  |  |  |  |  |  |
| **Log (10) IL-10** | -12.630 | 8.150 | -0.064 | 0.122 | -28.643-3.382 |
| Cohort | 15.474 | 13.104 | 0.099 | 0.238 | -10.272-41.220 |
| Gender | -48.119 | 13.574 | -0.193 | <0.001 | -74.789—21.450 |
| Age (years) | -0.310 | 2.611 | -0.010 | 0.905 | -5.441-4.820 |
| Percentage body fat (%) | -2.324 | 0.763 | -0.160 | 0.002 | -3.824- -0.825 |
| Weekly physical activity (24-h MET) | 4.483 | 0.981 | 0.195 | <0.001 | 2.557- 6.410 |
|  |  |  |  |  |  |
| **Log (10) IFN-γ** | -0.947 | 13.214 | -0.003 | 0.943 | -26.910- 25.016 |
| Cohort | 16.996 | 13.085 | 0.109 | 0.195 | -8.712- 42.704 |
| Gender | -47.442 | 13.554 | -0.191 | <0.001 | -74.071- -20.813 |
| Age (years) | -0.201 | 2.609 | -0.006 | 0.939 | -5.327- 4.926 |
| Percentage body fat (%) | -2.254 | 0.762 | -0.156 | 0.003 | -3.751- -0.757 |
| Weekly physical activity (24-h MET) | 4.478 | 0.978 | 0.196 | <0.001 | 2.556- 6.400 |
|  |  |  |  |  |  |
| **Log (10) TNF-alpha** | -40.287 | 26.913 | -0.063 | 0.135 | -93.164- 12.590 |
| Cohort | 16.661 | 13.038 | 0.107 | 0.202 | -8.955- 42.277 |
| Gender | -50.867 | 13.756 | -0.204 | <0.001 | -77.894- -23.841 |
| Age (years) | 0.042 | 2.610 | 0.001 | 0.987 | -5.086- 5.170 |
| Percentage body fat (%) | -2.144 | 0.765 | -0.148 | 0.005 | -3.647- -0.641 |
| Weekly physical activity (24-h MET) | 4.430 | 0.975 | 0.193 | <0.001 | 2.514- 6.346 |

*Linear regression analysis with GW/MFI-20 reduced motivation fatigue score as dependent variable. Sig.: p-value <0.05*

**Table A.17. Linear regression on CPV- reduced motivation ratio for per persons without inflammatory conditions**

| **Variables** | **Unstandardized B** | **S.E.** | **Standardized B** | **Sig.** | **95% confidence interval** |
| --- | --- | --- | --- | --- | --- |
| **Log (10) hs-CRP** | -38.659 | 16.166 | -0.135 | 0.017 | -70.448- -6.870 |
| Cohort | 18.879 | 16.315 | 0.110 | 0.248 | -13.203- 50.961 |
| Gender | -74.003 | 17.306 | -0.305 | <0.001 | -108.034- -39.971 |
| Age (years) | 0.556 | 3.098 | 0.017 | 0.858 | -5.535- 6.648 |
| Percentage body fat (%) | 0.963 | 1.141 | 0.060 | 0.399 | -1.281- 3.207 |
| Weekly physical activity (24-h MET) | 2.656 | 1.419 | 0.094 | 0.062 | -0.134- 5.445 |
|  |  |  |  |  |  |
| **Log (10) IL-6** | -52.003 | 20.426 | -0.135 | 0.011 | -92.170- -11.836 |
| Cohort | 19.008 | 16.309 | 0.111 | 0.245 | -13.062- 51.078 |
| Gender | -66.733 | 16.553 | -0.275 | <0.001 | -99.284- -34.183 |
| Age (years) | 1.389 | 3.123 | 0.041 | 0.657 | -4.751- 7.529 |
| Percentage body fat (%) | 0.457 | 1.056 | 0.029 | 0.665 | -1.619- 2.533 |
| Weekly physical activity (24-h MET) | 2.378 | 1.416 | 0.084 | 0.094 | -0.407- 5.163 |
|  |  |  |  |  |  |
| **Log (10) IL-10** | -21.752 | 10.890 | -0.102 | 0.047 | -43.167- -0.338 |
| Cohort | 19.206 | 16.370 | 0.112 | 0.241 | -12.985- 51.396 |
| Gender | -58.592 | 16.379 | -0.241 | <0.001 | -90.801- -26.382 |
| Age (years) | 1.134 | 3.131 | 0.034 | 0.717 | -5.023- 7.291 |
| Percentage body fat (%) | -0.398 | 1.023 | -0.025 | 0.697 | -2.409- 1.613 |
| Weekly physical activity (24-h MET) | 2.343 | 1.422 | 0.083 | 0.100 | -0.454- 5.139 |
|  |  |  |  |  |  |
| **Log (10) IFN-γ** | -44.867 | 16.487 | -0.138 | 0.007 | -77.288- -12.446 |
| Cohort | 24.572 | 16.453 | 0.413 | 0.136 | -7.782- 56.927 |
| Gender | -64.658 | 16.356 | -0.266 | <0.001 | -96.822- -32.495 |
| Age (years) | 1.524 | 3.123 | 0.046 | 0.623 | -4.60- 7.676 |
| Percentage body fat (%) | -0.086 | 1.017 | -0.005 | 0.933 | -2.087- 1.914 |
| Weekly physical activity (24-h MET) | 2.608 | 1.413 | 0.092 | 0.066 | -0.171- 5.388 |
|  |  |  |  |  |  |
| **Log (10) TNF-alpha** | -46.834 | 35.073 | -0.068 | 0.183 | -115.802- 22.133 |
| Cohort | 20.884 | 16.541 | 0.122 | 0.208 | -11.642- 53.410 |
| Gender | -62.836 | 16.518 | -0.259 | <0.001 | -95.317- -30.355 |
| Age (years) | 0.937 | 3.151 | 0.028 | 0.766 | -5.260- 7.134 |
| Percentage body fat (%) | -0.204 | 1.024 | -0.013 | 0.842 | -2.218- 1.810 |
| Weekly physical activity (24-h MET) | 2.374 | 1.422 | 0.084 | 0.096 | -0.423- 5.170 |

*Linear regression analysis with GW/MFI-20 reduced motivation fatigue score as dependent variable. Sig.: p-value <0.05*

**Table A.18. Linear regression on CPV- mental fatigue ratio for per persons with inflammatory conditions**

| **Variables** | **Unstandardized B** | **S.E.** | **Standardized B** | **Sig.** | **95% confidence interval** |
| --- | --- | --- | --- | --- | --- |
| **Log (10) hs-CRP** | -19.687 | 12.281 | -0.075 | 0.110 | -43.817- 4.443 |
| Cohort | 28.203 | 14.087 | 0.173 | 0.046 | 0.525- 55.881 |
| Gender | -76.609 | 15.017 | -0.294 | <0.001 | -106.144- -47.103 |
| Age (years) | 1.509 | 2.835 | 0.045 | 0.595 | -4.061- 7.078 |
| Percentage body fat (%) | -0.510 | 0.894 | -0.033 | 0.569 | -2.269- 1.247 |
| Weekly physical activity (24-h MET) | 2.585 | 1.046 | 0.108 | 0.014 | 0.529- 4.641 |
|  |  |  |  |  |  |
| **Log (10) IL-6** | -22.883 | 15.792 | -0.065 | 0.148 | -53.911- 8.145 |
| Cohort | 28.812 | 13.986 | 0.177 | 0.040 | 1.333- 56.291 |
| Gender | -77.339 | 14.782 | -0.298 | <0.001 | -106.382- -48.297 |
| Age (years) | 1.861 | 2.820 | 0.055 | 0.510 | -3.679- 7.401 |
| Percentage body fat (%) | -0.566 | 0.841 | -0.037 | 0.501 | -2.217- 1.086 |
| Weekly physical activity (24-h MET) | 2.547 | 1.04 | 0.107 | 0.015 | 0.502- 4.591 |
|  |  |  |  |  |  |
| **Log (10) IL-10** | -14.854 | 8.868 | -0.072 | 0.088 | -31.920- 2.213 |
| Cohort | 28.193 | 14.050 | 0.173 | 0.045 | 0.588- 55.797 |
| Gender | -73.383 | 14.459 | -0.284 | <0.001 | -102.248- -45.429 |
| Age (years) | 1.479 | 2.821 | 0.044 | 0.600 | -4.063- 7.021 |
| Percentage body fat (%) | -0.943 | 0.813 | -0.062 | 0.247 | -2.540- 0.654 |
| Weekly physical activity (24-h MET) | 2.641 | 1.044 | 0.110 | 0.012 | 0.589- 4.692 |
|  |  |  |  |  |  |
| **Log (10) IFN-γ** | -3.749 | 14.099 | -0.011 | 0.790 | -31.451- 23.952 |
| Cohort | 29.011 | 14.056 | 0.178 | 0.040 | 1.395- 56.627 |
| Gender | -72.782 | 14.466 | -0.280 | <0.001 | -101.205- -44.359 |
| Age (years) | 1.602 | 2.824 | 0.048 | 0.571 | -3.946- 7.149 |
| Percentage body fat (%) | -0.873 | 0.813 | -0.058 | 0.283 | -2.471- 0.724 |
| Weekly physical activity (24-h MET) | 2.637 | 1.044 | 0.110 | 0.012 | 0.586- 4.688 |
|  |  |  |  |  |  |
| **Log (10) TNF-alpha** | -31.043 | 28.715 | -0.047 | 0.280 | -87.460- 25.374 |
| Cohort | 29.241 | 14.002 | 0.180 | 0.037 | 1.704- 56.723 |
| Gender | -75.526 | 14.680 | -0.291 | <0.001 | -104.368- -46.684 |
| Age (years) | 1.808 | 2.823 | 0.054 | 0.522 | -3.739- 7.355 |
| Percentage body fat (%) | -0.783 | 0.816 | -0.052 | 0.338 | 2.386- 0.821 |
| Weekly physical activity (24-h MET) | 2.594 | 1.041 | 0.109 | 0.013 | 0.549- 4.638 |

*Linear regression analysis with GW/MFI-20 mental fatigue score as dependent variable. Sig.: p-value <0.05*

**Table A.19. Linear regression on CPV- mental fatigue ratio for per persons without inflammatory conditions**

| **Variables** | **Unstandardized B** | **S.E.** | **Standardized B** | **Sig.** | **95% confidence interval** |
| --- | --- | --- | --- | --- | --- |
| **Log (10) hs-CRP** | -40.954 | 17.932 | -0.128 | 0.023 | -73.216- -5.692 |
| Cohort | 24.677 | 18.097 | 0.128 | 0.174 | -10.910- 60.264 |
| Gender | -100.130 | 19.197 | -0.369 | <0.001 | -137.879- -62.381 |
| Age (years) | 0.431 | 3.436 | 0.011 | 0.900 | -6.326- 7.188 |
| Percentage body fat (%) | 2.168 | 1.266 | 0.122 | 0.088 | -0.322- 4.657 |
| Weekly physical activity (24-h MET) | 3.415 | 1.574 | 0.108 | 0.031 | 0.320- 6.509 |
|  |  |  |  |  |  |
| **Log (10) IL-6** | -67.537 | 22.538 | -0.157 | 0.003 | -111.858- -23.217 |
| Cohort | 25.044 | 17.995 | 0.131 | 0.165 | -10.343- 60.430 |
| Gender | -93.739 | 18.265 | -0.346 | <0.001 | -12.655- -57.822 |
| Age (years) | 1.615 | 3.445 | 0.043 | 0.640 | -5.16- 8.390 |
| Percentage body fat (%) | 1.812 | 1.15 | 0.102 | 0.121 | -0.478- 4.103 |
| Weekly physical activity (24-h MET) | 3.146 | 1.563 | 0.100 | 0.045 | 0.072- 6.219 |
|  |  |  |  |  |  |
| **Log (10) IL-10** | -18.512 | 12.083 | -0.078 | 0.126 | -42.272- 5.248 |
| Cohort | 24.738 | 18.163 | 0.129 | 0.174 | -10.979- 60.456 |
| Gender | -83.631 | 18.174 | -0.308 | <0.001 | -119.369- -47.893 |
| Age (years) | 0.931 | 3.474 | 0.025 | 0.789 | -5.900- 7.762 |
| Percentage body fat (%) | 0.762 | 1.135 | 0.043 | 0.502 | -1.469- 2.993 |
| Weekly physical activity (24-h MET) | 3.151 | 1.578 | 0.100 | 0.047 | 0.049- 6.254 |
|  |  |  |  |  |  |
| **Log (10) IFN-γ** | -40.454 | 18.338 | -0.111 | 0.028 | -76.514- -4.393 |
| Cohort | 29.669 | 18.300 | 0.154 | 0.106 | -6.318- 65.655 |
| Gender | -89.521 | 18.193 | -0.330 | <0.001 | -125.295- -53.746 |
| Age (years) | 1.269 | 3.474 | 0.034 | 0.715 | -5.561- 8.100 |
| Percentage body fat (%) | 1.027 | 1.132 | 0.058 | 0.365 | -1.198- 3.252 |
| Weekly physical activity (24-h MET) | 3.331 | 1.572 | 0.105 | 0.035 | 0.240- 6.423 |
|  |  |  |  |  |  |
| **Log (10) TNF-alpha** | -43.35 | 38.905 | -0.057 | 0.260 | -120.438- 32.569 |
| Cohort | 26.449 | 18.348 | 0.138 | 0.150 | -9.361- 62.529 |
| Gender | -87.975 | 18.323 | -0.324 | <0.001 | -124.005- -51.945 |
| Age (years) | 0.754 | 3.496 | 0.020 | 0.829 | -6.119- 7.628 |
| Percentage body fat (%) | 0.923 | 1.136 | 0.052 | 0.417 | -1.310- 3.157 |
| Weekly physical activity (24-h MET) | 3.119 | 1.577 | 0.099 | 0.049 | 0.017- 6.221 |

*Linear regression analysis with GW/MFI-20 mental fatigue score as dependent variable. Sig.: p-value <0.05*

**Table A.20 Gender -and cohort-specific cutoff values used for HIGH self-perceived fatigue and LOW muscle endurance**

P70 = percentile 70, P30 = percentile 30

| Parameter | MC | DALWUH | | CPC | |
| --- | --- | --- | --- | --- | --- |
|  | **Males** | **Males** | **Females** | **Males** | **Females** |
|  | *n=159* | *n=37* | *n=56* | *n=311* | *n=366* |
| *Cutoff values for HIGH self perceived fatigue* | ***P70*** | ***P70*** | ***P70*** | ***P70*** | ***P70*** |
| Total Fatigue score (score 20-100) | 52 | 44 | 50 | 44 | 48 |
| General Fatigue score (score 4-20) | 12 | 12 | 11 | 10 | 12 |
| Physical Fatigue score (score 4-20) | 12 | 11 | 11 | 10 | 12 |
| Reduced Activity score (score4-20) | 10 | 9 | 10 | 8 | 9 |
| Reduced motivation score (score 4-20) | 9 | 8 | 9 | 8 | 8 |
| Mental Fatigue score (score 4-20) | 10 | 8 | 9 | 9 | 10 |
| *Cutoff values for LOW muscle ecndurance* | ***P30*** | ***P30*** | ***P30*** | ***P30*** | ***P30*** |
| Grip Work /body mass (kg*sec*kg^-1^) | 11.5 | 13.0 | 6.5 | 12.6 | 9.1 |
